# Identification of Extracellular Matrix Signatures as Novel Potential Prognostic Biomarkers in Lung Adenocarcinoma

**DOI:** 10.3389/fgene.2022.872380

**Published:** 2022-05-30

**Authors:** Zhen Zeng, Yuanli Zuo, Yang Jin, Yong Peng, Xiaofeng Zhu

**Affiliations:** ^1^ Key Laboratory of Bio-Resource and Eco-Environment of Ministry of Education, College of Life Sciences, Sichuan University, Chengdu, China; ^2^ Laboratory of Molecular Oncology, Frontiers Science Center for Disease-related Molecular Network, State Key Laboratory of Biotherapy, West China Hospital, Sichuan University, Chengdu, China

**Keywords:** ECM, LUAD, TCGA, prognostic model, gene signature

## Abstract

The extracellular matrix (ECM) is vital to normal cellular function and has emerged as a key factor in cancer initiation and metastasis. However, the prognostic and oncological values of ECM organization-related genes have not been comprehensively explored in lung adenocarcinoma (LUAD) patients. In this study, we included LUAD samples from The Cancer Genome Atlas (TCGA, training set) and other three validation sets (GSE87340, GSE140343 and GSE115002), then we constructed a three-gene prognostic signature based on ECM organization-related genes. The prognostic signature involving *COL4A6*, *FGA* and *FSCN1* was powerful and robust in both the training and validation datasets. We further constructed a composite prognostic nomogram to facilitate clinical practice by integrating an ECM organization-related signature with clinical characteristics, including age and TNM stage. Patients with higher risk scores were characterized by proliferation, metastasis and immune hallmarks. It is worth noting that high-risk group showed higher fibroblast infiltration in tumor tissue. Accordingly, factors (*IGFBP5*, *CLCF1* and *IL6*) reported to be secreted by cancer-associated fibroblasts (CAFs) showed higher expression level in the high-risk group. Our findings highlight the prognostic value of the ECM organization signature in LUAD and provide insights into the specific clinical and molecular features underlying the ECM organization-related signature, which may be important for patient treatment.

## Introduction

Lung cancer remains the leading cause of cancer death worldwide, accounting for ∼11.4% of all new cancer cases and 18.0% of all cancer deaths (Sung et al., 2021). Non-small-cell lung cancer (NSCLC) accounts for ∼85% of lung cancers and has a poor 5-year survival rate. Lung adenocarcinoma (LUAD) is the most common pathological subtype of NSCLC and accounts for ∼40% of NSCLC cases (Piperdi et al., 2014). Surgical resection offers only the possibility for a cure at present. However, most LUAD patients are diagnosed at the metastasis stage. Although recent progress in targeted therapy and molecular pathology has facilitated clinical therapy, the 5-year overall survival (OS) rate of patients with LUAD remains low. To date, the tumor-node-metastasis (TNM) staging system is the gold standard for assessing prognosis and evaluating treatment results (Greene and Sobin, 2008). The high heterogeneity of LUAD leads to different outcomes among patients with the same TNM stage. Hence, it is imperative to develop individualized treatments and predict outcomes for patients with LUAD.

The extracellular matrix (ECM) regulates development and maintains tissue homeostasis (Mammoto and Ingber, 2010). Tumors often present desmoplasia, which is characterized by an alteration of ECM (Lu et al., 2012). Cancer-associated ECM can actively contribute to its histopathology and behaviors (Levental et al., 2009). For example, patients with pancreatic cancer exhibit marked stromal desmoplasia, which is often associated with tumor progression and poor outcome (Pandol et al., 2009). Breast cancer patients with high expression of matrix remodeling genes such as MMPs (Matrix Metalloproteinases) and collagen cross-linkers often have poor prognosis (Erler et al., 2006). Similarly, lung tumors showed ECM remodeling with high levels of hydroxylysine aldehyde-derived collagen cross-links and lower levels of lysine aldehyde-derived cross-links (Chen et al., 2015). Given that ECM alterations can contribute to a series of abnormalities, an ECM based individualized prediction of survival for patients with LUAD needs to be achieved.

In this study, we used four different LUAD cohorts, including RNA sequencing (RNA-seq) and microarray data, to construct and validate the ECM organization-related prognosis signature. We further established a composite prognostic nomogram to enhance clinical practice by integrating the ECM organization-related prognosis signature with clinical characteristics (age and tumor stage). In addition, the functional impact underlying the ECM organization-related prognostic signatures was explored between the high-risk and low-risk groups.

## Materials and Methods

### Lung Adenocarcinoma Data Source

We systematically searched public gene expression data and complete clinical annotation in TCGA and Gene Expression Omnibus (GEO) databases. Four LUAD cohorts with both expression data and clinical information (sex, age, TNM stage and prognosis data) available were finally included in this study (Supplementary Table S1), including TCGA-LUAD (443 LUAD and 53 normal samples), two sets of RNA sequencing data, GSE140343 (51 LUAD and 49 normal samples) and GSE87340 (23 LUAD and 23 normal samples), and microarray data GSE115002 (52 LUAD and 52 normal samples) (Supplementary Table S2).

### Data Preprocessing

For the high-throughput sequencing data from TCGA-LUAD and GEO datasets (GSE140343 and GSE87340), raw read count values were transformed into transcripts per kilobase million (TPM) values, which are more similar to those generated from microarrays. For the microarray data, the Agilent probe ID from the microarray was annotated to gene symbols according to the GPL13497 platform. For multiple probes that map to the same gene, the mean expression value was calculated. The ensemble ID for mRNAs from high-throughput sequencing data was transformed to gene symbols *via* the biomaRt package (Durinck et al., 2005). The final expression value for each dataset was given in log_2_(TPM+1), and the batch effect in each dataset was initially identified with box plot, then the normalizeBetweenArrays function in limma (Ritchie et al., 2015) was performed to remove batch effects in each dataset in which the samples showed distribution of difference.

### Differential Gene Expression Analysis Between LUAD and Normal Samples

DESeq2 (Love et al., 2014) was used to perform DGE analysis between LUAD and normal samples for each dataset. Genes were selected as differentially expressed genes based on the statistical threshold (|log_2_FoldChange| > 1 and adjusted *p* value < 0.05), here log_2_FoldChange = mean(log_2_(LUAD)—mean(log_2_(normal samples))). Then, the overlapping differentially expressed genes of the four datasets were obtained, and Gene ontology (GO) enrichment was performed on these genes with clusterProfile package (Wu et al., 2021) in R.

### Collection of ECM Organization Related Genes

The GO enrichment results show ECM organization (GO:0030198) was the top enrichment signature (Supplementary Figure S1). ECM organization-related genes, defined as genes related to a process that is carried out at the cellular level that results in the assembly, arrangement of constituent parts, or disassembly of external structures that lie outside the plasma membrane and surround the entire cell, were collected from the GO term (GO:0030198) in the AmiGO database (Carbon et al., 2009). ECM organization-related genes shared among the eligible LUAD cohorts were retained for further studies.

### Identification of ECM Organization-Related Prognostic Signatures

Univariate Cox proportional hazards regression analysis was first performed on the expression matrix of ECM organization-related genes to estimate the relationship between these genes and OS in the LUAD samples of the TCGA-LUAD cohort. ECM organization-related genes with a *p* value < 0.01 were selected as potential prognosis-related genes. Then, the LASSO (least absolute shrinkage and selection operator) penalty (Tibshirani, 1997) was performed with glmnet package (Simon et al., 2011) in the discovery cohort to build an optimal prognostic signature with the minimal number of ECM organization-related genes. Tenfold cross-validation was conducted to tune the optimal value of penalty parameter λ, which yielded the minimum partial likelihood deviance. Then, a set of prognostic signature candidates and their nonzero coefficients were identified. The correlated variables were further removed, and finally multivariate Cox proportional hazards regression analysis was performed on the remaining ECM organization-related prognosis signature candidates. A signature with a *p* value < 0.05 was selected for the final candidates with independent prognostic potential. The genes that met the conditions were further subjected to multivariate Cox proportional hazards regression together with one or more potential signatures. Both the univariate and multivariate Cox analyses were performed with coxph function in survival package. And the final risk score for the selected signature was calculated for each sample based on the formula:

$$\text{R}\text{i}\text{s}\text{k}\;\text{s}\text{c}\text{o}\text{r}\text{e}=\sum_{i=1}^{n}Coef_{i}\times E_{i}$$

Where Coef_i_ is the coefficient and E_i_ is the normalized expression value of each selected signature by log_2_ transformation. The corresponding coefficients derived from the TCGA-LUAD cohort were then used in the other three validation datasets. Patients were dichotomized into high-risk and low-risk groups using the best cutoff measured by receiver operator characteristic (ROC) curves with pROC package (Robin et al., 2011) for both training data and validation datasets (GEO datasets). The performance of the signature model was evaluated by time-dependent ROC curves with survivalROC package (Heagerty et al., 2000). The performance of risk groups determined by risk scores was assessed based on the overall survival time difference between the high-risk and low-risk groups. Kaplan–Meier curves were generated for survival rates, with distance detection based on log-rank testing.

### Development of a Composite ECM Organization-Clinical Prognostic Nomogram

The patients in the high-risk group of TCGA-LUAD cohorts were further divided into three groups according to the risk scores, and then multivariate regression analysis was performed on the risk groups and clinical characteristics (age, sex, TNM stage and smoking status). Based on the multivariate analysis results, we integrated age, TNM stage and the ECM organization-related prognostic signature to generate a composite prognostic model by applying a Cox proportional hazard regression in the TCGA-LUAD cohort. Then, a nomogram was generated for model visualization and clinical application. The performance of the nomogram was evaluated by time-dependent ROC analysis and calibration curves.

### Immunohistochemical Analysis

Protein expression data were obtained from the Human Protein Atlas (HPA) database, which is the largest and most comprehensive database for evaluating protein distribution in human tissues (Thul and Lindskog, 2018). The protein expression of the selected prognostic genes related to ECM organization in normal and lung cancer was determined using immunohistochemical staining images. HPA064755 and HPA005723 are antibodies for FGA and FSCN1 respectively.

### Gene Set Enrichment Analysis

Based on the risk scores, the LUAD samples in TCGA-LUAD dataset were divided into high-risk and low-risk groups as mentioned above. Then, DGE analysis between high-risk and low-risk group was performed with DESeq2, and a pre-ranked list sorted by log2FoldChange was generated to perform GSEA (Subramanian et al., 2005), here log2FoldChange = mean(log2(samples of high-risk group)—mean(log2(low-risk group))). The Molecular Signatures Database (MSigDB) is a collection of annotated gene sets for GSEA software use (https://www.gsea-msigdb.org/gsea/msigdb/index.jsp) (Liberzon et al., 2015). Significant differences were demonstrated in the hallmark gene sets of MSigDB (h.all.v7.2.symbols. GMT) collection (Liberzon et al., 2015).

### Weighted Correlation Network Analysis

In order to obtain the signature-related modules, WGCNA (Langfelder and Horvath, 2008) was performed on LUAD samples in TCGA-LUAD dataset. The gene module associated with ECM organization-related prognosis was identified using WGCNA according to the protocol and recommendations of the WGCNA package. The top 5,000 most variant genes measured by the median absolute deviation (MAD) were screened for WGCNA performance. A scale-free topology fitting index *R* ^2^ > 0.9 was set as the threshold to construct the weighted gene coexpression network. A biweight midcorrelation coefficient (r) ≥ 0.3 and *p* value < 0.05 were set as the thresholds for determining gene modules associated with the prognostic signatures (Age, tumor stage, overall survival time (day) and risk score).

### Immune Heterogeneity Analysis

The presence of infiltrating stromal and immune cells in tumors of TCGA-LUAD cohorts was estimated with estimate package (Yoshihara et al., 2013). The population abundance of tissue infiltrating immune and stromal cell populations was assessed with MCPcounter package (Becht et al., 2016).

## Results

### Overview of ECM Organization Related Genes in LUAD

A total of 752 samples from four independent datasets (LUAD-TCGA, GSE140343, GSE87340, GSE115002) were collected, including 569 LUAD samples and 183 normal adjacent samples (Supplementary Table S2). First, DGE analysis was conducted between LUAD and normal samples across each dataset. Then, GO enrichment was performed on the shared differentially expressed genes, and the results showed that ECM organization was the top enrichment signature (Supplementary Figure S1), implying that ECM indeed plays an important role in LUAD tumorigenesis and development. Therefore, we decided to explore the prognostic potential of ECM organization-related genes. Then, all related ECM organization (GO: 0030198) terms were collected, 344 (Supplementary Table S3) of which were present in all datasets. The expression profiles of these genes between LUAD and normal samples in each dataset are shown in Supplementary Figure S2.

### Identification of ECM Organization-Related Prognostic Signatures

**FIGURE 1:**
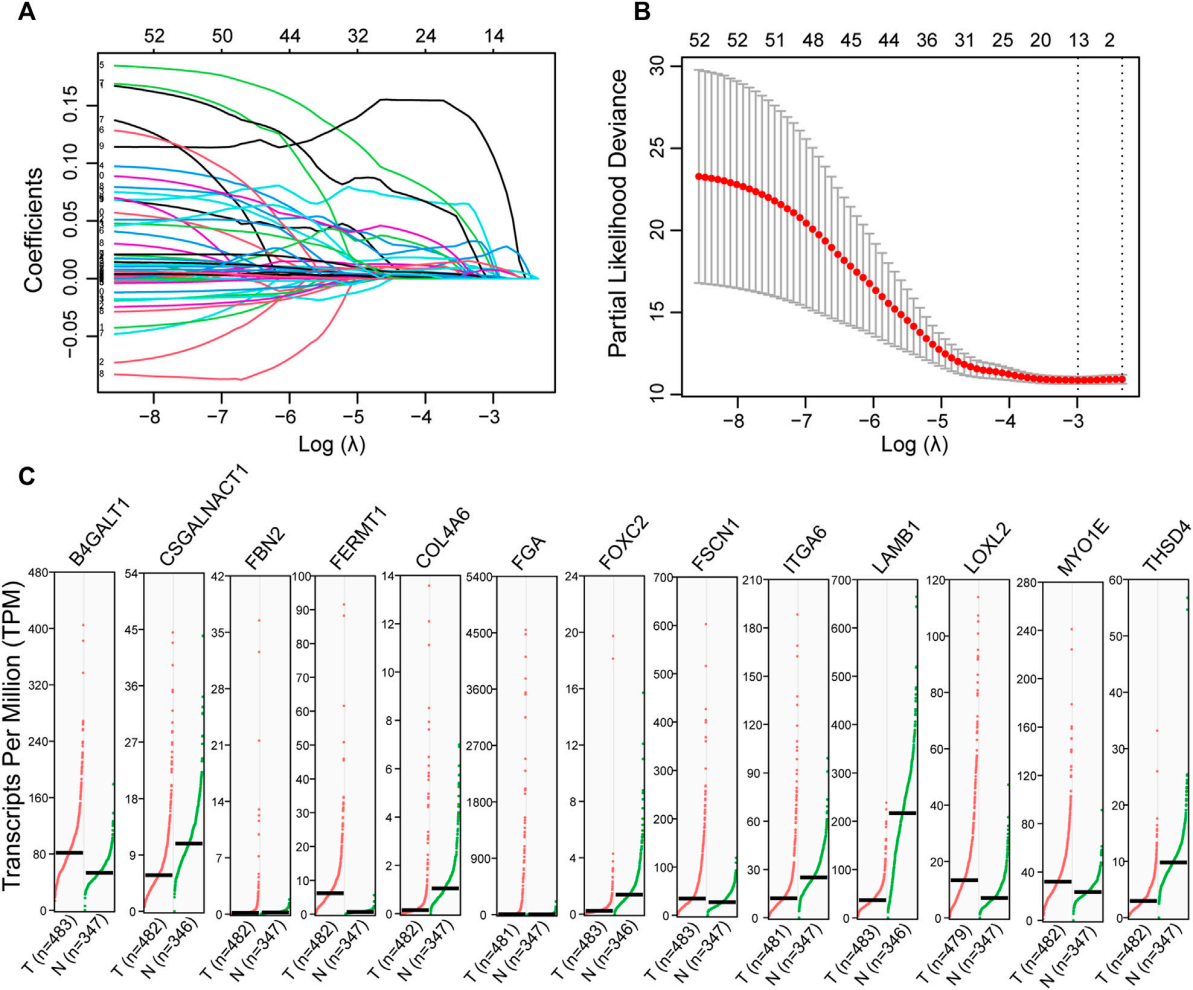
Identification of the ECM organization-related prognostic signature. **(A)** The changing trajectory of every single gene identified with univariate Cox proportional hazards analysis. **(B)** The confidence interval at different value of λ. **(C)** The expression level of potential prognostic candidates in LUAD (GEPIA). These genes were selected by Cox regression with LASSO. Dot plots profiling gene expression between LUAD (red dots) and normal adjacent lung tissues (green dots), with each dot representing a sample. The TPM value was used to display the relative expression of these genes.

Of 344 ECM organization-related genes, 54 were associated with OS (Supplementary Table S4). The LASSO Cox regression algorithm was applied to perform feature selection (Figures 1A,B), and 13 ECM organization-related genes were retained. The expression patterns of these genes were illustrated with GEPIA (http://gepia.cancer-pku.cn/index.html) using LUAD samples from TCGA compared to both TCGA and GTEx (Genome Tissue Expression) normal samples (Figure 1C). The results suggested that most of these genes were dysregulated in LUADs (*n* = 483) compared with normal samples (*n* = 347).

Then, the correlated variable (*FOXC2*) was removed, multivariate Cox proportional hazards regression analysis was performed on the remaining related prognosis signature candidates, and the signature with *p* value <0.05 was selected for the final candidates with independent prognostic potential (Table 1). The genes that met the conditions were further subjected to multivariate Cox proportional hazards regression together with one or more potential signatures. Three genes were ultimately used to establish an ECM organization-related signature (Table 1). The corresponding risk scores were computed for both the training and validation datasets according to the following formula:

Risk score=0.263⁡exp(COL4A6)+0.0232⁡exp(FSCN1)+0.0037⁡exp(FGA)

**TABLE 1:** Univariate and multivariate Cox analysis of 12 prognosis related ECM organization genes.

Genes	Univariate analysis			Multivariate analysis		
	HR	*p*-value	CI 95	HR	*p*-value	CI 95
B4GALT1	1.02	0.000173	1.009–1.03	-	-	-
FERMT1	1.065	0.000937	1.026–1.106	-	-	-
COL22A1	1.148	0.009639	1.034–1.275	-	-	-
COL4A6	1.264	0.000302	1.113–1.436	1.301	0.000249	1.130–1.498
CSGALNACT1	1.152	0.000158	1.07–1.24	-	-	-
FBN2	1.058	2.47E-05	1.031–1.086	-	-	-
FGA	1.003	0.0082	1.001–1.005	1.004	0.000462	1.002–1.006
FSCN1	1.022	5.86E-07	1.013–1.03	1.023	7.17E-08	1.015–1.032
ITGA6	1.019	0.00031	1.009–1.03	-	-	-
LOXL2	1.034	1.35E-07	1.021–1.047	-	-	-
LAMB1	1.036	5.28E-05	1.019–1.055	-	-	-
MYO1E	1.059	0.000406	1.026–1.094	-	-	-

**FIGURE 2:**
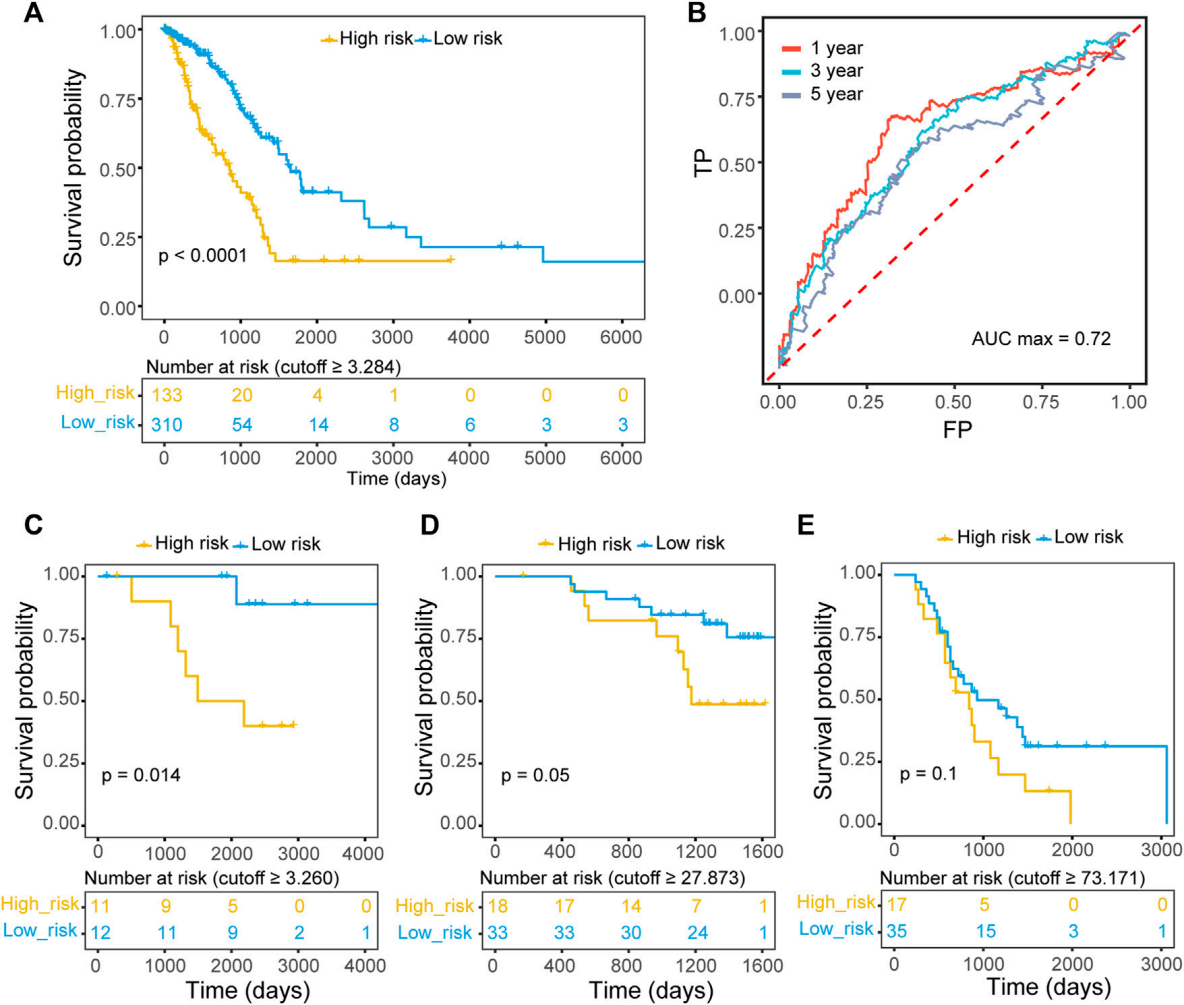
The prognostic value of the ECM organization-related three-gene signature. **(A)** Kaplan–Meier OS curves with difference detection by log-rank test for patients from training data (TCGA-LUAD). Patients were divided into two groups according to the best cutoff measured by ROC curve. **(B)** Prognostic value of the ECM organization-related three-gene signature evaluated by time-dependent ROC curves (1-, 3- and 5-year) in TCGA-LUAD datasets. The AUC for 1-year survival was 0.72. **(C–E)** Kaplan–Meier OS curves with difference detection by log-rank test for patients from three validation datasets, GSE87340 **(C)**, GSE140343 **(D)** and GSE115002 **(E)**.

The patients in the TCGA-LUAD training set were divided into high-risk and low-risk groups according to the best cutoff (risk score cutoff = 3.284) measured by ROC curve analysis. Kaplan–Meier survival analysis determined that patients with lower risk scores had significantly longer OS than those with higher risk scores (*p* value < 0.00001; Figure 2A). ROC curves were utilized to evaluate the predictive power, and the best area under the curve (AUC) was 0.72 for 1-, 3-, and 5-year OS (Figure 2B). Consistently, patients in three validation datasets were divided into two groups with different cutoffs estimated by ROC curves (GSE87340: risk score cutoff = 3.260; GSE140343: risk score cutoff = 27.873; GSE115002: risk score cutoff = 73.171), and patients with lower risk scores had significantly longer OS (GSE87340: *p* value = 0.014; GSE140343: *p* value = 0.05; GSE115002: *p* value = 0.1; Figures 2C–E).

### Identification of Composite Prognostic Nomogram

**FIGURE 3:**
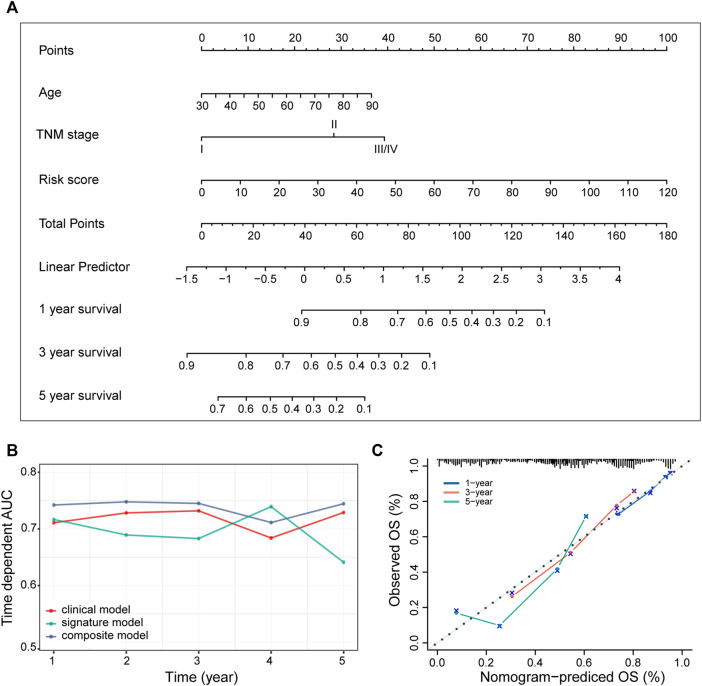
Identification of the composite prognostic nomogram. **(A)** Composite nomogram prediction of 1-, 3-, and 5-year OS. **(B)** Time-dependent AUC of ROC curves for the nomogram, signature model and clinical data at different time points in the TCGA-LUAD dataset. **(C)** Calibration curves of observed and predicted probabilities for the nomogram in the TCGA-LUAD dataset.

In addition to the ECM organization-related signature, clinical characteristics such as age and TNM stage might also be independent prognostic signatures (Table 2), which implies their complementary value. These clinical variables were integrated with the 3-gene signature to further improve the prognostic accuracy using the coefficients generated from the multivariate Cox regression model in the TCGA-LUAD cohort and derived a composite prognostic model. A nomogram was then established for model visualization and clinical application (Figure 3A). The composite nomogram performed better than both the ECM organization-related prognostic signature model and clinical model (Figure 3B). The calibration curve detected an optimal prediction between the nomogram prediction and actual observation (Figure 3C).

**TABLE 2:** Multivariate Cox analysis of clinical characteristics and risk groups.

Factor	HR	CI 95	*p*-value
Age	1.03	1.01–1.1	**0.004**
Gender (male vs. female)	0.85	0.55–1.3	0.45
Tumor stage			
II vs. I	2.91	1.77–4.8	**< 0.001**
III vs. I	3.63	2.18–6.0	**< 0.001**
IV vs. I	3.73	1.84–7.6	**< 0.001**
Risk group			
High first vs. Low	2.96	1.71–5.1	**< 0.001**
High second vs. Low	2.04	1.05–3.9	**0.035**
High third vs. Low	3.06	1.75–5.3	**< 0.001**
smoke status (Non-smoking vs. Smoking)	1.31	0.68–2.5	0.423

The p-value with bold means the outcome is statistically significant.

### Expression and Clinical Features Underlying the ECM Organization-Related Prognostic Signature

**FIGURE 4:**
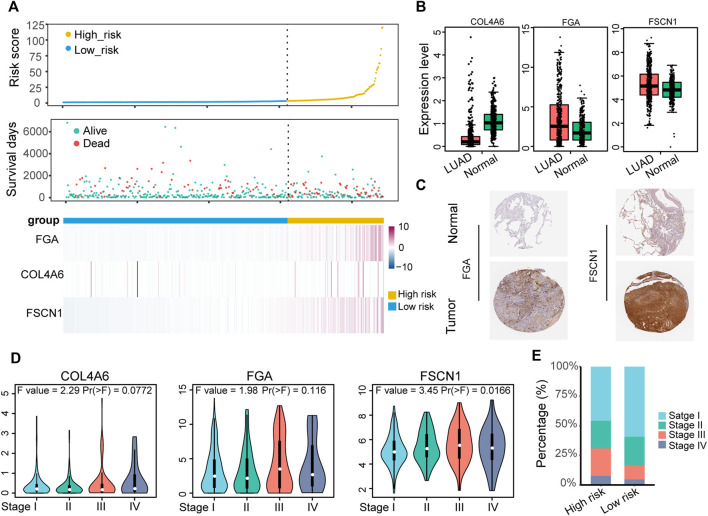
Identification of the expression and clinical features underlying the ECM organization-related prognostic signature. **(A)** The distribution of survival status and expression profile of the three prognostic-associated ECM organization genes for the TCGA-LUAD dataset sorted by signature risk score in ascending order. **(B)** The expression level of the three signature genes in LUAD (GEPIA). **(C)** Immunohistochemical analysis of the protein expression of FGA and FSCN1 in LUAD and normal lung tissues in the HPA database. **(D)** Expression level of three signatures in different TNM stages of LUAD. **(E)** Histogram showing the distribution of TNM stages between low-risk and high-risk groups.

LUAD samples in the TCGA-LUAD cohort were pooled to explore the expression and clinical features of the ECM organization-related prognostic signatures. The distribution of the survival status and expression profile of *COL4A6* (collagen type IV alpha 6 chain), *FGA* (fibrinogen alpha chain) and *FSCN1* (fascin actin-bunding protein 1) between the high-risk and low-risk groups is presented in Figure 4A. All three of these genes were risk-associated genes, as they showed higher expression levels in patients with higher risk scores. *COL4A6* was lower in LUAD samples than in normal samples (Figure 4B), and the other two genes showed higher RNA expression in LUAD samples, as well as the protein expression level (Figures 4B,C). However, the expression levels of all three markers showed an increasing tendency during the tumor TNM stage (Figure 4D). Patients with advanced tumor stage (stage III and stage IV) were significantly enriched in the high-risk group (Figure 4E). *COL4A6* (collagen type IV alpha 6 chain) is a member of the COL4A family, a major component of the basement membrane (BM), which may be involved in tumor angiogenesis and progression (Socovich and Naba, 2019). Ikeda *et al.* showed that *COL4A6* was also downregulated in colorectal cancer compared with normal colorectal tissues and it might remodel the epithelial BM during cancer cell invasion (Ikeda et al., 2006). However, the expression level of *COL4A6* slightly increased with TNM stage, which may imply its different roles in the tumor environment and needs to be further explored.

### Function Analysis of Genes Correlated With ECM Organization Related Prognostic Signature

**FIGURE 5:**
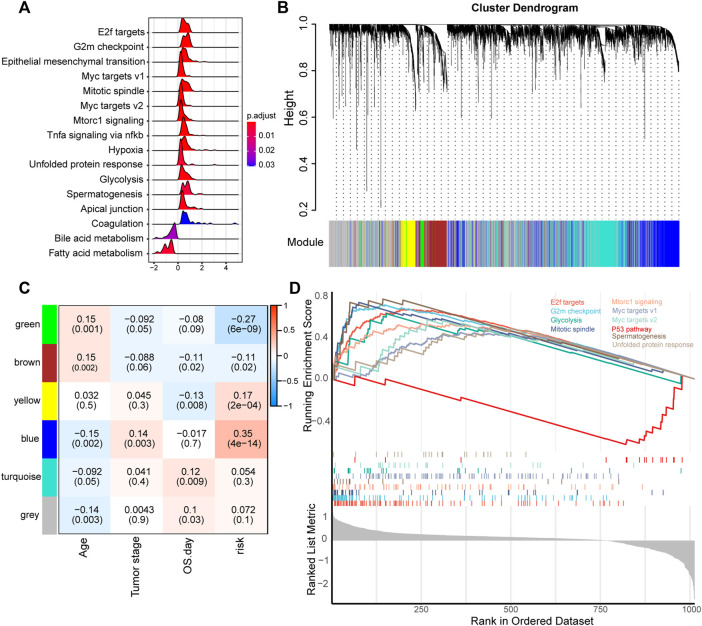
Functional analysis of genes correlated with the ECM organization-related prognostic signature. **(A)** GSEA of the hallmark gene set for risk groups based on pre-ranked fold change between high-risk and low-risk groups calculated by DESeq2. **(B)** Clustering dendrogram of the top 5,000 variant genes with dissimilarity based on the topological overlap together with assigned module colors. **(C)** Module-trait relationships. Each row represents a module eigengene, each column corresponds to a clinical trait, and each cell contains the corresponding correlation (upper number) and *p* value (lower number). **(D)** GSEA of the hallmark gene set of genes in the blue module.

Given that ECM plays an important role in cancer progression, we subsequently evaluated the mRNA expression profile influenced by the ECM organization-related prognostic signatures. We first preranked the genes according to their fold changes between high-risk and lower-risk groups calculated by DESeq2, then we performed GSEA (Subramanian et al., 2005). The results indicated that proliferation, metastasis and immune hallmarks, such as E2F targets, G2M checkpoint, Myc targets, EMT and TNFα signaling *via* NFKB were significantly enriched in LUAD samples with higher risk scores. In contrast, metabolism hallmarks, such as bile acid metabolism and fatty acid metabolism were enriched in LUAD samples with lower risk scores (Figure 5A).

Furthermore, we used WGCNA to obtain the signature-related modules according to the approximate scale-free features. The top 5,000 most variant genes measured by the median absolute deviation (MAD) were screened for WGCNA performance. We chose five as the optimal soft threshold power to calculate the adjacency matrix, which was the lowest threshold to enable the scale-free *R* ^2^ to reach 0.9 (Supplementary Figure S3). We then construct a cluster dendrogram with an adjacency matrix. Six color modules (yellow, blue, green, brown, turquoise and gray) were identified (Figure 5B). Genes that could not be included in any module were placed in the gray module.

Module-trait relationships between eigengenes of selected traits and modules were evaluated. The blue module was highly significantly associated with the high-risk group (|R| > 0.3) (Figure 5C). Functional enrichment analysis of genes in the blue module, pre-ranked according to DESeq2 analysis between the high-risk and low-risk groups mentioned above, was performed to explore the biological functions. The results suggested that the E2F targets, G2M checkpoint, and myc targets were significantly enriched in genes of the blue module (Figure 5D). These findings implied that the ECM organization-related prognostic signature reflects the expression alteration of genes involved in multiple cancer hallmarks in LUAD.

### Immune Heterogeneity Underlying the ECM Organization-Related Prognostic Signature

**FIGURE 6:**
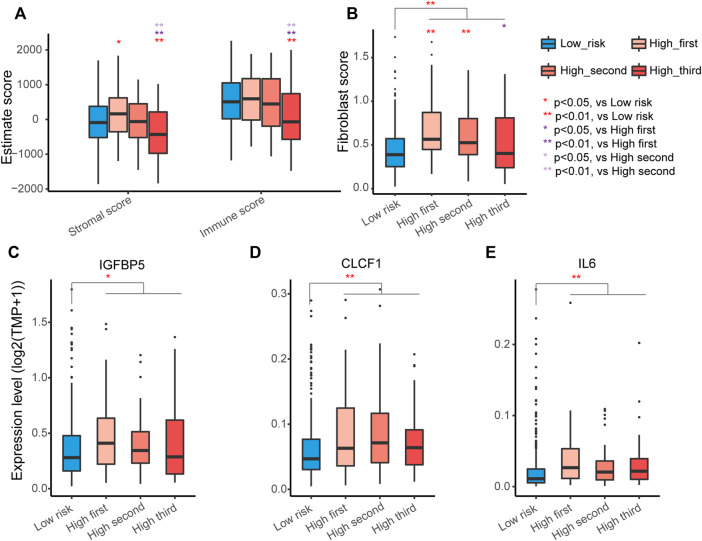
Identification of immune heterogeneity underlying the ECM organization-related prognostic signature. **(A)** Differential expression of immune stromal score and immune score among different groups. The high-risk group was further divided into three groups according to the risk scores. The asterisks represent the statistical *p* values (∗*p* < 0.05; ∗∗*p* < 0.01). **(B)** Differential expression of fibroblasts among different groups. **(C–E)** Different expression levels of factors secreted by the CAFs *IGFBP5* **(C)**, *CLCF1* **(D)** and *IL6* **(E)**.

The tumor microenvironment encompasses host stromal cells and noncellular components, including the ECM. Then, we explored the relationship between the tumor microenvironment status and the ECM organization-related signature to characterize their immune heterogeneity. The stromal and immune scores, representing stromal and immune cell infiltration status in tumor tissue, respectively, were estimated for each sample of LUAD in the TCGA-LUAD cohort. The results suggested that there was no difference in stromal and immune scores between low-risk and all high-risk samples, while stromal and immune scores decreased as risk scores increased in the high-risk groups (Figure 6A). The MCPcounter algorithm detected no difference in certain cells except for fibroblasts (Supplementary Figure S4), and patients with higher risk scores had a higher percentage of fibroblasts in tumor samples, especially in the high-first and high-second groups (Figure 6B).

Fibroblasts are the major components of the tumor microenvironment in most solid tumors, and activated cancer-associated fibroblasts (CAFs) play important roles in cancer development *via* their secretion of acellular components, such as ECM (Socovich and Naba, 2019). *IGFBP5*, one factor can be secreted by CAFs (Weigel et al., 2014), was significantly higher in all high-risk groups (Figure 6C). CAFs can also secrete the cytokines cardiotrophin-like cytokine factor 1 (*CLCF1*) and interleukin 6 (*IL6*) to directly stimulate the growth of tumor cells (Vicent et al., 2012). Indeed, *CLCF1* and *IL6* were significantly elevated in the high-risk groups (Figures 6D,E). These results indicated that the activation of fibroblasts in the tumor environment of LUAD likely contributes to the worse prognosis of patients with LUAD in the high-risk group.

## Discussion

Lung cancer remains the leading cause of cancer death worldwide. The high morbidity rate of lung cancer is due to tobacco smoking, genetic alteration, and outdoor and indoor air pollution (Hamra et al., 2014). Although recent progress in targeted therapy and molecular pathology has enhanced clinical therapy, the 5-year OS rate of LUAD patients remains low (Qi et al., 2016). Hence, further understanding of the molecular mechanisms underlying tumorigenesis and progression of LUAD may enhance the overall prognosis and treatment of this tumor.

The ECM is an important noncellular component that plays essential roles in the development and progression of cancer. Originally believed to be more of a static unit that maintains tissue integrity, it was later recognized that the ECM is vital to normal cellular function and has emerged as another key factor of cancer initiation and metastasis (Alexander and Friedl, 2012; Lu et al., 2012). In this study, we first constructed a three-gene ECM organization-related prognostic signature to predict the prognosis of stratified patients with LUAD. The identified signature was integrated with clinical features, including age and TNM stage, to establish the composite prognostic nomogram, which serves as a statistical tool with great clinical applications to more accurately assess the overall probability of specific outcomes for individual patients with LUAD.

COL4A6 is a risk-related gene in the three-gene signature model, and it is a member of the COL4A family, which is a major component of BM. BM acts as a physical barrier for prohibiting invasion and metastasis (Zeng et al., 2020). We found that *COL4A6* was downregulated in LUAD, while the expression level of COL4A6 slightly increased with TNM stage. Downregulation of COL4A6 could change BM constituents, making it possible for invasion or metastasis of tumor cells. However, given the robust prognostic potential of COL4A6, the impact of its increased expression on TNM stage may reveal the dynamics of ECM remodeling. Our results provide some open questions to be addressed: why did COL4A6 show lower expression in LUAD but increased expression with TNM stage? Does it play different roles in LUAD and normal lung tissues?

The functional impact underlying the ECM organization-related prognostic signatures was finally explored between the high-risk and low-risk groups, and fibroblasts were significantly infiltrated in the tumor tissue of LUAD patients with higher risk scores. Within the tumor stroma, not only cancer cells but also resident fibroblasts, which differentiate into cancer-associated fibroblasts (CAFs), modify the ECM. The ECM serves as a reservoir for a number of growth factors and cytokines, which are crucial for cell differentiation and proliferation (Taipale and Keski-Oja, 1997; Hynes and Naba, 2012). The factors (*IGFBP5*, *CLCF1* and *IL6*) reported to be secreted by CAFs indeed showed significantly higher expression in the high-risk group, suggesting that fibroblasts in the tumor microenvironment of LUAD likely contribute to the poor outcome in LUAD patients.

However, there are limitations in this study. First, the limited sample number in the validation datasets made it impossible to evaluate the prognostic value in each validation dataset. Second, further *in vitro* and *in vivo* experiments regarding these prognostic-related ECM organization genes are required to validate our findings.

In conclusion, our study highlights the prognostic value of ECM organization-related genes in LUAD and reveals an ECM organization-related prognostic signature for further improving the prognosis prediction of patients with LUAD with definite TNM stage. The functional impact underlying the signature was also explored. Our findings provide a basis for understanding the roles of these genes in ECM organization and indicate the potential clinical implications of these genes in LUAD.

## Data Availability

The original contributions presented in the study are included in the article/Supplementary Material, further inquiries can be directed to the corresponding authors.

## Supplementary Material

Supplementary Figure S1 GO enrichment of overlapping DEGs.

Supplementary Figure S2 Shared ECM organization related genes in each dataset. **(A)** TCGA-LUAD, **(B)** GSE87340, **(C)** GSE140343 and **(D)** GSE115002.

Supplementary Figure S3 Identification of the soft threshold according to the standard of the scale-free network. The red line represents the threshold of 0.90.

Supplementary Figure S4 Differential expression of immune and stromal cells among different risk groups.

Supplementary Table S1 Information of public datasets used in this study.

Supplementary Table S2 Clinical information of samples included in this study.

Supplementary Table S3 344 ECM organization related gene shared by datasets included this study.

Supplementary Table S4 54 OS associated ECM organization related gene.

Supplementary Script S1 R code for the construction of prognostic model based on ECM organization gene set.

Supplementary Script S2 R code for functional analysis between patients of different risk groups.
