# Supplementary material for: Identification of Extracellular Matrix Signatures as Novel Potential Prognostic Biomarkers in Lung Adenocarcinoma
Source: Front Genet. 2022 May 30;13:872380. doi: 10.3389/fgene.2022.872380 (PMC9197387; doi:10.3389/fgene.2022.872380)
Supplement: Supplementary file 1 [file DataSheet2.PDF]

```
## Code for functional analysis between patients of different risk groups
## infiltration of Immune cell and stroma cell ####
## estimate
library(estimate)
setwd("/survival_model/EMO/")
TCGA <- read.csv("../TCGA_LUAD_gene_count_Final.csv",check.names = FALSE)
rownames(TCGA) <- TCGA$geneID
colnames(TCGA)[1] <- "GeneSymbol"
colnames(TCGA) <- gsub("-","_",colnames(TCGA))
write.table(TCGA[,-1],"TCGA_LUAD_gene_count_Final.txt",sep="\t",quote=FALSE)


filterCommonGenes(input.f="TCGA_LUAD_gene_count_Final.txt",output.f =
'TCGA_LUAD_gene_count_Final1.gct')
estimateScore(input.ds = 'TCGA_LUAD_gene_count_Final1.gct',
              output.ds = 'ESTIMATE_score.gct',
              platform = "illumina")

ESTIMATE_score <- read.table('ESTIMATE_score.gct',
                             skip =2,
                             header=T,
                             row.names = 1) %>%
  as.matrix() %>% t() %>% as.data.frame()

rownames(ESTIMATE_score) <- gsub("_","-",rownames(ESTIMATE_score))

ESTIMATE_score$Sample <- rownames(ESTIMATE_score)
head(ESTIMATE_score)
df <- left_join(data3,ESTIMATE_score[-1,],by="Sample",all.x=TRUE)

df$StromalScore <- as.double(df$StromalScore)
df$ImmuneScore <- as.double(df$ImmuneScore)
df$ESTIMATEScore <- as.double(df$ESTIMATEScore)
plot_df <- dplyr::select(df,c("Sample","group","StromalScore","ImmuneScore")) %>%
  reshape2::melt(id.vars = c("Sample","group"),variable.name = "type",value.name="score")
head(plot_df)
ggplot(plot_df,aes(x=type,y=score,fill=group))+
  geom_boxplot(width=0.6,outlier.size = 0.3)+
  labs(x="",y="Estimate score",fill="")+
  theme_classic()+
  scale_fill_manual(values = c("#E7B800", "#2E9FDF")) +
  scale_x_discrete(limits = c("StromalScore","ImmuneScore"),
                   labels = c("Stromal score","Immune score"))+
  theme(axis.text.x = element_text(angle=30,hjust=1,vjust=1))
```

```
ggsave("16.Stromal_Immune_score_between_risk_groups.pdf",height=3,width=5)

wilcox.test(plot_df[plot_df$type=="StromalScore" & plot_df$group=="High_risk",]$score,
            plot_df[plot_df$type=="StromalScore" &
plot_df$group=="Low_risk",]$score,
            alternative = "greater")    ## p = 0.01596

wilcox.test(plot_df[plot_df$type=="ImmuneScore" & plot_df$group=="High_risk",]$score,
            plot_df[plot_df$type=="ImmuneScore" &
plot_df$group=="Low_risk",]$score,
            alternative = "greater") # p = 0.366

## MCP-counter
library(devtools)
# install_github("ebecht/MCPcounter",ref="master",subdir="Source")
library(MCPcounter)
TCGA_TPM <-
read.csv("../TCGA_LUAD_logTPM_normalizationBetweenArrays.csv",header=T,check.names =
FALSE)
rownames(TCGA_TPM) <- TCGA_TPM$geneID
MCPcounter_estimate <- MCPcounter.estimate(TCGA_TPM[,-1],featuresType =
"HUGO_symbols")
tmp    <- as.matrix(MCPcounter_estimate) %>% t() %>% as.data.frame()
tmp$Sample <- rownames(tmp)

df <- left_join(df,tmp,by="Sample")
head(df)
plot_df1 <- dplyr::select(df,c("Sample","group","T cells","CD8 T cells","Cytotoxic
lymphocytes",
                               "B lineage","NK cells","Monocytic
lineage","Myeloid dendritic cells",
                               "Neutrophils","Endothelial
cells","Fibroblasts")) %>%
  reshape2::melt(id.vars= c("Sample","group"),variable.name="type",value.name = "score")

ggplot(plot_df1[plot_df1$type=="Fibroblasts",],aes(x=group,y=score,fill=group))+
  geom_boxplot(width=0.5,outlier.size = 0.4)+
  labs(x="",y="Estimate score",fill="")+
  theme_classic()+
  scale_fill_manual(values = c("#E7B800", "#2E9FDF")) +
  geom_signif(comparisons = list(c("High_risk","Low_risk")),step_increase =
0.05,map_signif_level = T,
              test = t.test,size=0.2,textsize = 8,tip_length=0.02,color="black")+
  theme(legend.position = "none")
```

```
ggsave("17.Stromal_cell_between_risk_groups.pdf",height=4,width=3.5)

wilcox.test(plot_df1[plot_df1$type=="Fibroblasts" & plot_df1$group=="High_risk",]$score,
            plot_df1[plot_df1$type=="Fibroblasts" &
plot_df1$group=="Low_risk",]$score,
            alternative = "greater")    ## p = 3.101e-10

CAF_gene <-
TCGA_LUAD_TPM[TCGA_LUAD_TPM$geneID=="CLCF1"|TCGA_LUAD_TPM$geneID=="IL6"|

TCGA_LUAD_TPM$geneID=="CNTFR"|TCGA_LUAD_TPM$geneID=="IL6R"|
                              TCGA_LUAD_TPM$geneID=="IGFBP5",] %>%
  as.matrix() %>%
  t() %>%
  as.data.frame()

colnames(CAF_gene) <- c("CLCF1","CNTFR","IGFBP5","IL6","IL6R")
CAF_gene$Sample <- rownames(CAF_gene)

CAF_gene <- CAF_gene[-1,] %>%

left_join(df[,c("Sample","group","total_risk_score","Tumor_stage","OS","OS.day","Fibroblasts","
StromalScore")],by="Sample") %>%
  na.omit()

CAF_gene$CLCF1 <- as.double(CAF_gene$CLCF1)
CAF_gene$IL6 <- as.double(CAF_gene$IL6)

CAF_gene$CNTFR <- as.double(CAF_gene$CNTFR)
CAF_gene$IL6R <- as.double(CAF_gene$IL6R)

CAF_gene$IGFBP5 <- as.double(CAF_gene$IGFBP5)

ggplot(CAF_gene,aes(x=group,y=CLCF1,fill=group))+
  geom_boxplot(outlier.size = 0.3,width=0.7)+
  geom_signif(comparisons = list(c("High_risk","Low_risk")),step_increase =
0.05,map_signif_level = T,
              test = wilcox.test,size=0.2,textsize = 4,tip_length=0.01,color="black")+
  labs(x="",y="Expression level (log2(TMP+1))",title="CLCF1")+
  scale_x_discrete(limits=c("High_risk","Low_risk"),labels = c("High risk","Low risk"))+
  theme_classic()+
  scale_fill_manual(values = c("#E7B800", "#2E9FDF")) +
  theme(legend.position = "none",
        plot.title = element_text(hjust=0.5))
```

```
ggsave("18.CAF_related_gene_CLCF1.pdf",height=4,width=3)

ggplot(CAF_gene,aes(x=group,y=IL6,fill=group))+
    geom_boxplot(outlier.size = 0.3,width=0.7)+
    geom_signif(comparisons = list(c("High_risk","Low_risk")),step_increase =
0.05,map_signif_level = T,
                              test = wilcox.test,size=0.2,textsize = 4,tip_length=0.01,color="black")+
    labs(x="",y="Expression level (log2(TMP+1))",title="IL6")+
    scale_x_discrete(limits=c("High_risk","Low_risk"),labels = c("High risk","Low risk"))+
    theme_classic()+
    scale_fill_manual(values = c("#E7B800", "#2E9FDF")) +
    theme(legend.position = "none",
                plot.title = element_text(hjust=0.5))
ggsave("18.CAF_related_gene_IL6.pdf",height=4,width=3)

ggplot(CAF_gene,aes(x=group,y=CNTFR,fill=group))+
    geom_boxplot(outlier.size = 0.3,width=0.7)+
    geom_signif(comparisons = list(c("High_risk","Low_risk")),step_increase =
0.05,map_signif_level = T,
                              test = wilcox.test,size=0.2,textsize = 4,tip_length=0.01,color="black")+
    labs(x="",y="Expression level (log2(TMP+1))",title="CNTFR")+
    scale_x_discrete(limits=c("High_risk","Low_risk"),labels = c("High risk","Low risk"))+
    theme_classic()+
    scale_fill_manual(values = c("#E7B800", "#2E9FDF")) +
    theme(legend.position = "none",
                plot.title = element_text(hjust=0.5))

ggsave("18.CAF_related_gene_CNTFR.pdf",height=4,width=3)

ggplot(CAF_gene,aes(x=group,y=IL6R,fill=group))+
    geom_boxplot(outlier.size = 0.3,width=0.7)+
    geom_signif(comparisons = list(c("High_risk","Low_risk")),step_increase =
0.05,map_signif_level = T,
                              test = wilcox.test,size=0.2,textsize = 4,tip_length=0.01,color="black")+
    labs(x="",y="Expression level (log2(TMP+1))",title="IL6R")+
    scale_x_discrete(limits=c("High_risk","Low_risk"),labels = c("High risk","Low risk"))+
    theme_classic()+
    scale_fill_manual(values = c("#E7B800", "#2E9FDF")) +
    theme(legend.position = "none",
                plot.title = element_text(hjust=0.5))
ggsave("18.CAF_related_gene_IL6R.pdf",height=4,width=3)

ggplot(CAF_gene,aes(x=group,y=IGFBP5,fill=group))+
```

```
    geom_boxplot(outlier.size = 0.3,width=0.7)+
    geom_signif(comparisons = list(c("High_risk","Low_risk")),step_increase = 
0.05,map_signif_level = T,
                              test = wilcox.test,size=0.2,textsize = 4,tip_length=0.01,color="black")+
    labs(x="",y="Expression level (log2(TMP+1))",title="IGFBP5")+
    scale_x_discrete(limits=c("High_risk","Low_risk"),labels = c("High risk","Low risk"))+
    theme_classic()+
    scale_fill_manual(values = c("#E7B800", "#2E9FDF")) +
    theme(legend.position = "none",
              plot.title = element_text(hjust=0.5))

ggsave("18.CAF_related_gene_IGFBP5.pdf",height=4,width=3)

## GSEA ####
TCGA_count <- read.csv("../TCGA_LUAD_gene_count_Final.csv",check.names = 
FALSE,row.names = 1)
TCGA_count[1:5,1:5]
TCGA_count <- TCGA_count[,df$Sample]
sample_info <- data.frame(condition = as.factor(df$group))
sample_info

rownames(sample_info) <- df$Sample
sample_info1 <- sample_info
sample_info1$condition<- factor(sample_info1$condition,levels=c("Low_risk","High_risk"))
head(sample_info1)
head(sample_info)

diff_expr <- DESeqDataSetFromMatrix(countData = TCGA_count,
                                                    colData = sample_info1,design = 
~condition)

diff_expr <- DESeq(diff_expr)
diff_result <- results(diff_expr) %>% as.data.frame()

ranks <- dplyr::select(diff_result,log2FoldChange)
ranks$ID     <- rownames(ranks)
ranks <- arrange(ranks,log2FoldChange) %>% .[,c("ID","log2FoldChange")]

gene <- str_trim(ranks$ID,"both")
gene <- bitr(gene,fromType="SYMBOL",toType="ENTREZID",OrgDb = "org.Hs.eg.db")
gene <- dplyr::distinct(gene,SYMBOL,.keep_all = TRUE)
gene_df <- data.frame(logFC=ranks[gene$SYMBOL,]$log2FoldChange,
                                  SYMBOL=gene$SYMBOL) %>%
    left_join(gene,by="SYMBOL")
```

```
geneList <- gene_df$logFC
names(geneList)=gene_df$SYMBOL
geneList = sort(geneList,decreasing = T)
head(geneList)

gmt <- read.gmt("../04.Extracellular_matrix_organization-
1/09.GSEA/h.all.v7.2.symbols.gmt")
gmt$term <- sub('HALLMARK_','',gmt$term)
gmt$term <- tolower(gmt$term)
gmt$term <- gsub('_',' ',gmt$term)
gmt$term <- sapply(gmt$term,function(x) capitalize(as.character(x)))
head(gmt)
result <- GSEA(geneList, TERM2GENE     = gmt,pval=0.05)

pdf("19.GSEA_enrichment.pdf",height=7,width=10)
ridgeplot(result)+
  labs("enrichment distribution")
dev.off()

## WGCNA ####
library(reshape2)
library(stringr)
# BiocManager::install("WGCNA")
library(WGCNA)
enableWGCNAThreads(2)
options(stringsAsFactors = FALSE)
TCGA_TPM[1:5,1:5]
exprMat <- TCGA_TPM[,df$Sample]
WGCNA_matrix <- t(exprMat[order(apply(exprMat,1,mad),decreasing = T)[1:5000],])
dataExpr <- WGCNA_matrix

nGenes = ncol(dataExpr)
nSample = nrow(dataExpr)

dataExpr_tree <- hclust(dist(dataExpr),method="average")
pdf("20.Sample_tree.pdf",height=10,width=40)
plot(dataExpr_tree,main="Sample clutering",sub="",xlab="",cex.lab=2,
     cex.axis = 1, cex.main = 1,cex.lab=1)
dev.off()

powers = c(c(1:10),seq(from=12,to=20,by=2))
powers
```

```
sft = pickSoftThreshold(dataExpr,RsquaredCut = 0.9,powerVector = powers,verbose=5)

pdf("20.beta_value.pdf",width=10,height=7)
par(mfrow=c(1,2))
cex1=0.9
plot(sft$fitIndices[,1],-sign(sft$fitIndices[,3])*sft$fitIndices[,2],
        xlab = "Soft Threshold (power)",ylab= "Scales free topology Fit, signed R^2",
        type="n",main="Scale independence")
text(sft$fitIndices[,1],-sign(sft$fitIndices[,3])*sft$fitIndices[,2],
        labels =powers,cex=0.9,col="red")
abline(h=0.90,col="red")

plot(sft$fitIndices[,1],sft$fitIndices[,5],
        xlab = "Soft threshold (power)",ylab = "Mean Connectivity",type="n",
        main = "Mean connectivity")
text(sft$fitIndices[,1],sft$fitIndices[,5],labels=powers,cex=cex1,col="red")
dev.off()

net = blockwiseModules(dataExpr,power = sft$powerEstimate,
                       maxBlockSize = 5000,
                       networkType = "unsigned",
                       TOMType = "unsigned",
                       deepSplit = 0,
                       minModuleSize = 40,
                       mergeCutHeight = 0.4,
                       pamRespectsDendro = FALSE,
                       saveTOMs = TRUE,
                       saveTOMFileBase = "log2TPM-TOM",
                       loadTOM = TRUE,
                       verbose=3)
table(net$colors)

pdf("20.gene_module.pdf",height=8,width=12)
plotDendroAndColors(net$dendrograms[[1]],net$colors,
                    "Module colors",
                    dendroLabels = FALSE,
                    hang=0.03,
                    addGuide = TRUE,guideHang = 0.05)
dev.off()

nGenes = ncol(dataExpr)
nSamples = nrow(dataExpr)

## module ME
```

```
MEs0 <- moduleEigengenes(dataExpr[,net$goodGenes],
                                            net$colors)$eigengene
MEs <- orderMEs(MEs0)
rownames(MEs) <- rownames(dataExpr[,net$goodGenes])
text <- cbind(rownames(MEs),MEs)
head(text)
colnames(text) <- "Sample"
write.csv(text,"20.module_eigengens.csv",quote=FALSE,row.names=F)

#
names(MEs) <- substring(names(MEs),3)
MEDiss <- 1-cor(MEs)
METree <- hclust(as.dist(MEDiss),method="average")
plot(METree,main="Clustering of module eigengenes",xlab="",sub="")
# correlation among modules
moduleCor <- corAndPvalue(MEs,use="p")
rowLabels <- paste("ME",names(MEs),sep="")
textMatrix <- paste(signif(moduleCor$cor,2),"\n(",signif(moduleCor$p,1),")",sep="")
dim(textMatrix) <- dim(moduleCor$cor)
pdf("20module_relationship.pdf",height=8,width=9)
labeledHeatmap(Matrix=moduleCor$cor,
                         textMatrix=textMatrix,
                         xLabels=rowLabels,
                         yLabels=rowLabels,
                         xSymbols=names(MEs),
                         ySymbols=names(MEs),
                         colorLabels=TRUE,
                         colors=blueWhiteRed(50),
                         setStdMargins=FALSE,
                         xLabelsAngle=90,zlim=c(-1,1))
dev.off()
text <- paste("cor=",round(moduleCor$cor,4),";p-value=",round(moduleCor$p,4),sep="")
dim(text) <- dim(moduleCor$cor)
rownames(text) <- rowLabels
colnames(text) <- rowLabels
text    <- cbind(rownames(text),text)
colnames(text)[1] <- "modules"
write.csv(text,"20.module_relationship.csv",row.names = F)

## ME and node gene
dir.create("expression_ME")
setwd("expression_ME/")
for(module in colnames(MEs)){
  pdf(paste0("express_ME_",module,".pdf"),width=40,height=15)
```

```
    ME <- t(as.matrix(MEs[,module]))
    colnames(ME) <- rownames(dataExpr[,net$goodGenes])
    layout(matrix(c(1,2)),heights=c(1.5,3))
    par(mar=c(0.3,9,3,5))
    plotMat(t(scale(dataExpr[,net$goodGenes][,net$colors==module])),
                    nrgcols=30,rlabels=F,rcols=module,
                    main=paste(module),cex.main=1)
    par(mar=c(5,4,0,1))
    barplot(ME,col=module,main="",cex.names=1,cex.axis=1,
                    ylab="module eigengene",las=3)
    dev.off()
}

## correlation between module and trait
dataTrait <- df[,c("Sample","Age","Tumor_stage","OS.day","total_risk_score")]

dataTrait$Tumor_stage[dataTrait$Tumor_stage=="I"] <- 1
dataTrait$Tumor_stage[dataTrait$Tumor_stage=="II"] <- 2
dataTrait$Tumor_stage[dataTrait$Tumor_stage=="III"] <- 3
dataTrait$Tumor_stage[dataTrait$Tumor_stage=="IV"] <- 4


rownames(dataTrait) <- dataTrait$Sample

moduleTraitCor <- cor(MEs,dataTrait[rownames(MEs),-1],use="p")

nSamples <- nrow(dataExpr[,net$goodGenes])
moduleTraitPvalue <- corPvalueStudent(moduleTraitCor,nSamples)
textMatrix <- paste(signif(moduleTraitCor,2),
                                        "\n(",signif(moduleTraitPvalue,1),")",sep="")
dim(textMatrix) <- dim(moduleTraitCor)
rowLabels <- paste("ME",names(MEs),sep="")
setwd("../")
pdf(file="20.modules_traits_relationships.pdf",width=6,height=6.5)
labeledHeatmap(Matrix=moduleTraitCor,
                              textMatrix=textMatrix,
                              xLabels=colnames(dataTrait)[-1],
                              yLabels=rowLabels,
                              ySymbols=names(MEs),
                              colorLabels=TRUE,
                              colors=blueWhiteRed(50),
                              setStdMargins=FALSE,
                              xLabelsAngle=90,zlim=c(-1,1))
dev.off()
```

```
text <- paste0("cor=",round(moduleTraitCor,4),";p-value=",round(moduleTraitPvalue,4))
dim(text) <- dim(moduleTraitCor)
rownames(text) <- rownames(moduleTraitCor)
colnames(text) <- colnames(moduleTraitCor)
text <- cbind(rownames(text),text)
colnames(text)[1] <- "modules"
write.csv(text,file="20.modules_traits_relationships.csv",
          row.names=F)

#
modNames <- names(MEs)
geneModuleMembership <- cor(dataExpr[,net$goodGenes],MEs,use="p")
nSamples <- nrow(dataExpr[,net$goodGenes])
MMPvalue <- corPvalueStudent(geneModuleMembership,nSamples)
colnames(geneModuleMembership) <- paste("MM",modNames,sep="")
colnames(MMPvalue) <- paste0("p.MM",modNames)
text <- paste0("cor=",round(geneModuleMembership,4),
           ";p-value=",round(MMPvalue,4))
dim(text) <- dim(geneModuleMembership)
write.csv(text,file="20.genes_module_membership.xls",row.names=F)

#
modNames <- names(MEs)
risk <- as.data.frame(dataTrait$total_risk_score)
names(risk) <- "risk"
geneTraitSignificance <- as.data.frame(cor(dataExpr[,net$goodGenes],risk,use="p"))

GSPvalue <- as.data.frame(corPvalueStudent(as.matrix(geneTraitSignificance),nSamples))
names(geneTraitSignificance) <- paste("GS.",names(risk),sep="")
names(GSPvalue) <- paste("p.GS.",names(risk),sep="")
head(geneTraitSignificance)
dir.create("20.MM_vs_risk")
setwd("20.MM_vs_risk/")

for(module in colnames(MEs)) {
  column <- match(module,modNames)
  moduleGenes <- names(net$colors[net$colors==module])
  jpeg(file=paste("07.",module,"_MM_vs_risk.jpeg",sep=""),600,600)
  verboseScatterplot(geneModuleMembership[moduleGenes,column],
                     geneTraitSignificance[moduleGenes,1],
                     xlab=paste("Module membership (MM) in",module,"module"),
                     ylab="Gene significance for risk",
                     main=paste("Module membership vs. gene significance\n"),
```

```
                                        col=module)
    dev.off()
}

##
setwd("../")
text <- cbind(geneTraitSignificance,GSPvalue)
text <- cbind(rownames(text),text)
colnames(text)[1] <- "genes"
write.csv(text,file="20.genes_trait_significance.csv",row.names=F)

## turquoise module
dir.create("20.turquoise_module")
setwd("./20.turquoise_module/")
turquoise_gene <- names(net$colors[net$colors=="turquoise"])

turquoise_gene_ranks <- ranks[turquoise_gene,]
gene <- str_trim(turquoise_gene_ranks$ID,"both")
gene <- bitr(gene,fromType="SYMBOL",toType="ENTREZID",OrgDb = "org.Hs.eg.db")
gene <- dplyr::distinct(gene,SYMBOL,.keep_all = TRUE)
gene_df <- data.frame(logFC=ranks[gene$SYMBOL,]$log2FoldChange,
                                        SYMBOL=gene$SYMBOL) %>%
    left_join(gene,by="SYMBOL")


geneList <- gene_df$logFC
names(geneList)=gene_df$SYMBOL
geneList = sort(geneList,decreasing = T)
head(geneList)

result <- GSEA(geneList, TERM2GENE     = gmt,pval=0.05)
pdf("20.turquoise_GSEA_enrichment.pdf",height=7,width=10)
ridgeplot(result)+
    labs("enrichment distribution")
dev.off()


library(ReactomePA)
library(enrichplot)
pdf("20.GSEA_enrichment_multiple.pdf",height=12,width=14)
gseaplot2(result,1:10,pvalue_table = TRUE,
                    color=pal_npg("nrc", alpha =0.9)(10))
dev.off()
getwd()
```
