# Supplementary figures and images for: Identification of Extracellular Matrix Signatures as Novel Potential Prognostic Biomarkers in Lung Adenocarcinoma
Source: Front Genet. 2022 May 30;13:872380. doi: 10.3389/fgene.2022.872380 (PMC9197387; doi:10.3389/fgene.2022.872380)

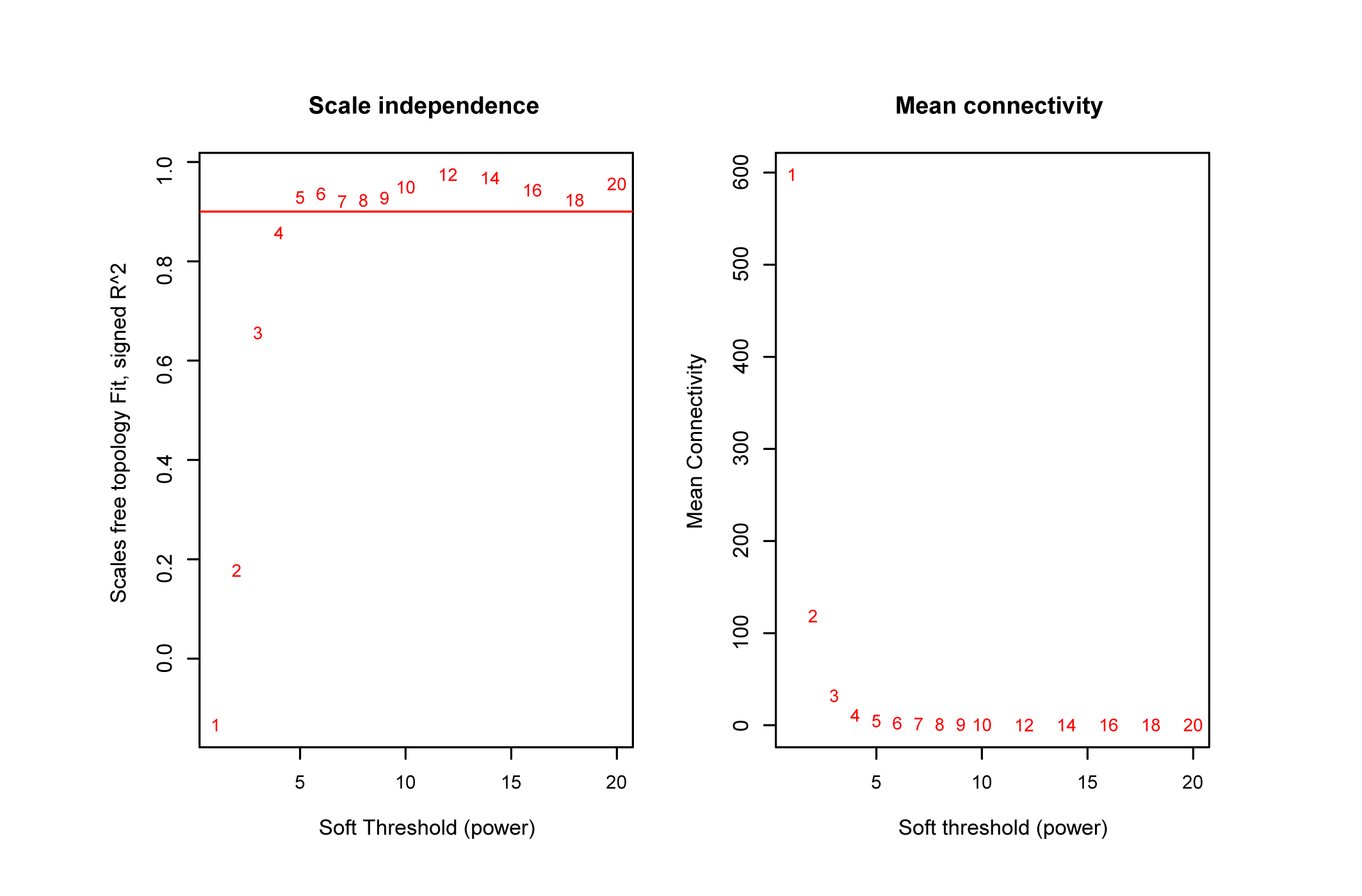

Supplement: Supplementary file 4 [file Image3.TIF]

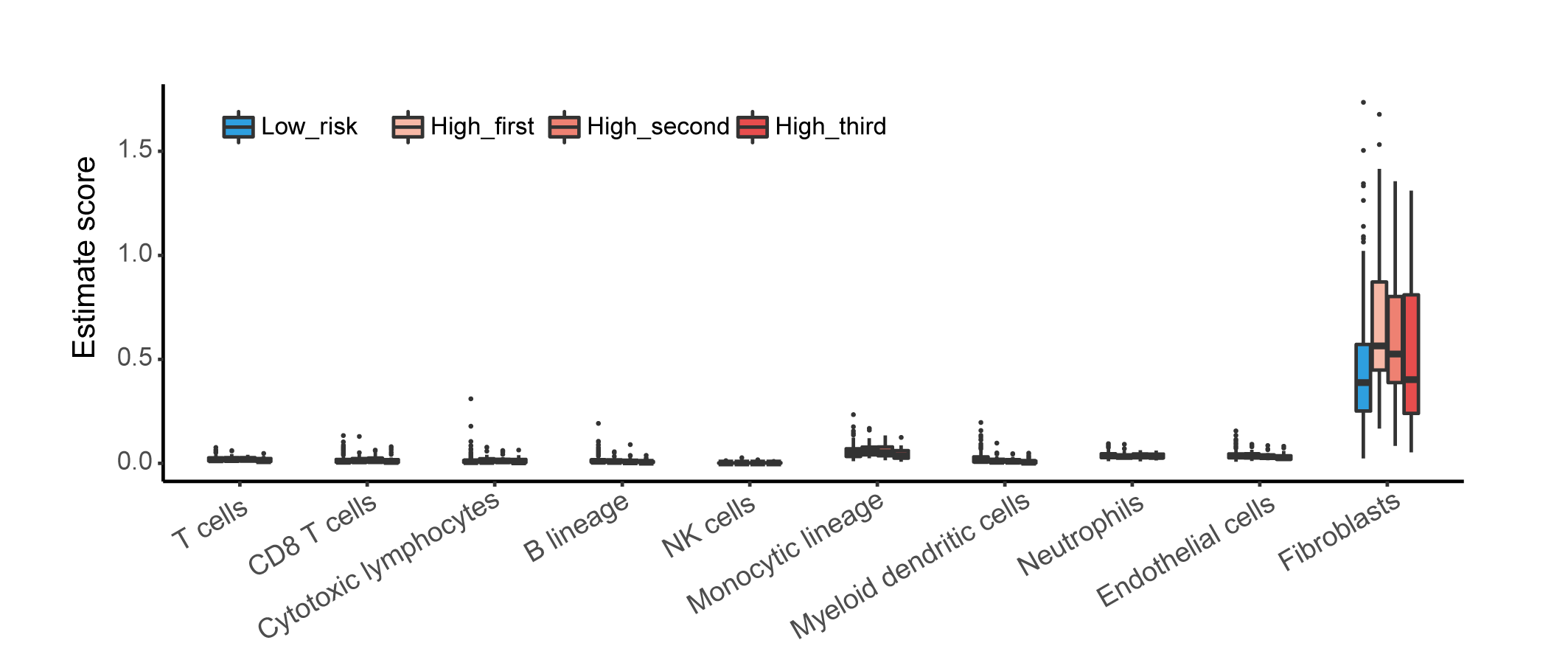

Supplement: Supplementary file 5 [file Image4.TIF]

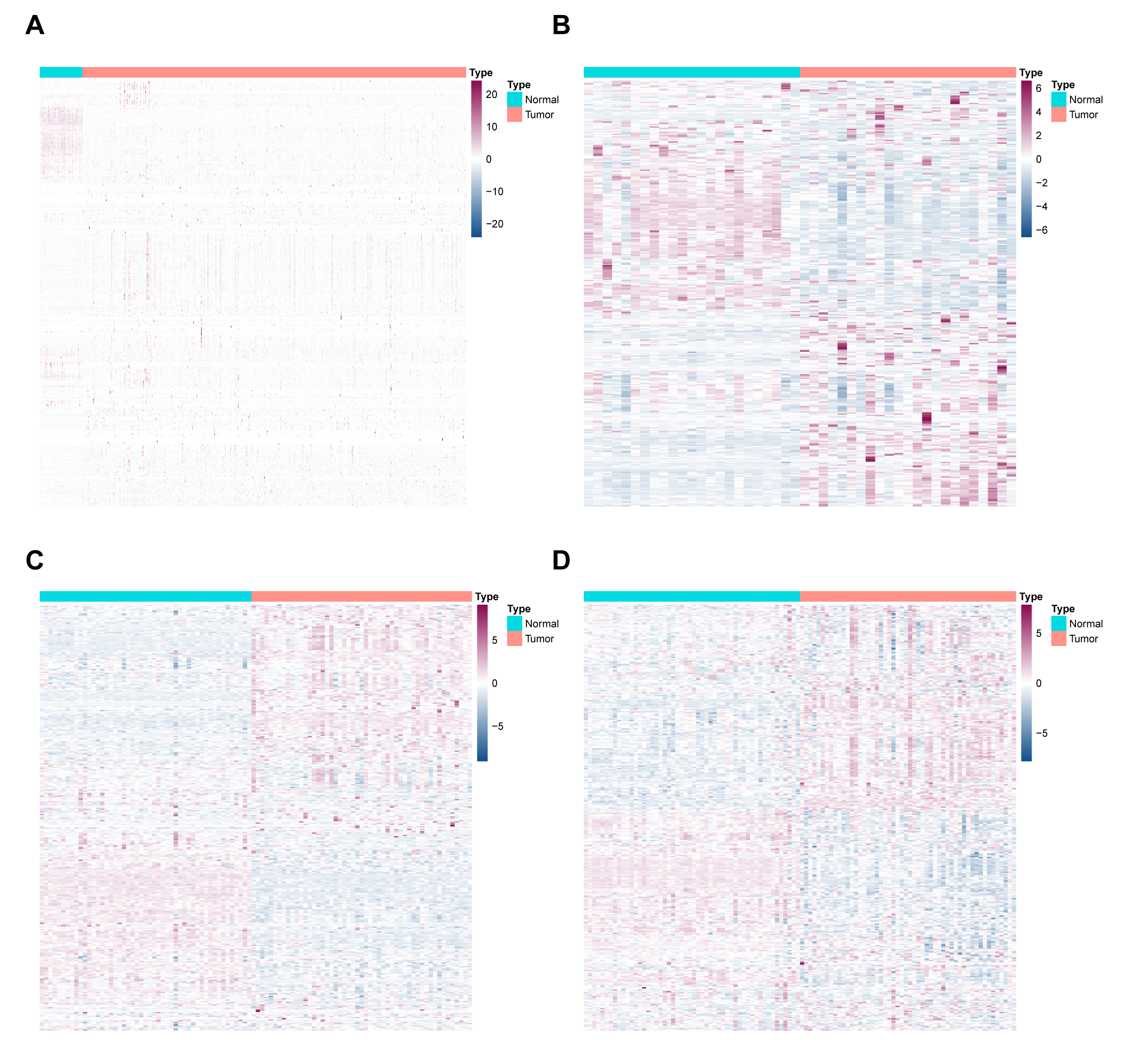

Supplement: Supplementary file 6 [file Image2.TIF]

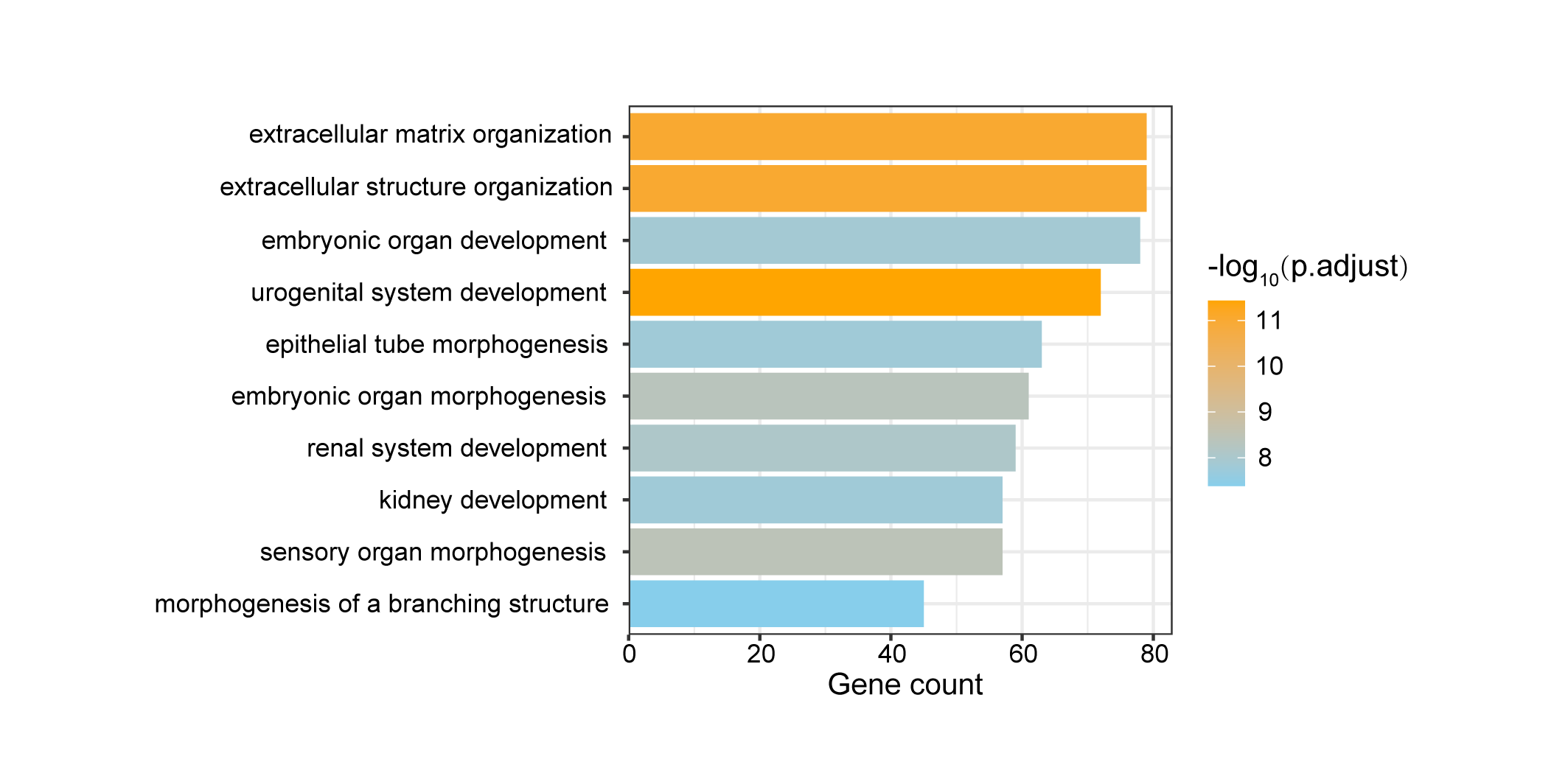

Supplement: Supplementary file 7 [file Image1.TIF]
