# Supplementary material for: Identification of Extracellular Matrix Signatures as Novel Potential Prognostic Biomarkers in Lung Adenocarcinoma
Source: Front Genet. 2022 May 30;13:872380. doi: 10.3389/fgene.2022.872380 (PMC9197387; doi:10.3389/fgene.2022.872380)
Supplement: Supplementary file 8 [file DataSheet1.PDF]

```
## R code for the construction of prognostic model based on ECM organization gene set
suppressMessages(library(UCSCXenaTools))
suppressMessages(library(dplyr))
library(survival)
library(limma)
library(survminer)
library(plyr)
library(dplyr)
library(reshape2)
library(ggplot2)
library("ggsci")
library(ggrepel)
library(scales)
library(gplots)
library(pheatmap)
# BiocManager::install("ConsensusClusterPlus")
#library(ConsensusClusterPlus)

#BiocManager::install("biomaRt")
library(biomaRt)
mypal =pal_npg("nrc", alpha =0.7)(10)
show_col(mypal)

setwd("./survival_model/01.log2TPM/")
## All
All_sample_info <- read.csv("../04.Extracellular_matrix_organization-1/Sample_clinical_information.csv",header=T,check.names = FALSE)

All_sample_info$Sample
head(All_sample_info)
All_sample_info$Tumor_stage <- sub("A","",All_sample_info$Tumor_stage)
All_sample_info$Tumor_stage <- sub("B","",All_sample_info$Tumor_stage)
All_sample_info$Tumor_stage <- sub("C","",All_sample_info$Tumor_stage)

All_sample_info$Tumor_stage <- sub("1","",All_sample_info$Tumor_stage)
All_sample_info$Tumor_stage <- sub("2","",All_sample_info$Tumor_stage)
All_sample_info$Tumor_stage <- sub("3","",All_sample_info$Tumor_stage)
unique(All_sample_info$Tumor_stage)


## dataSet TPM ####
## GSE115002
GSE115002_expr <- read.csv("../GSE115002_expr_Final.csv",header=T,check.names = FALSE)
```

```
ggplot(GSE115002_expr %>%
melt(id.vars=c("geneID"),variable.name="Sample",value.name = "TPM"),
            aes(x = Sample,y = log(TPM+1))) +
   geom_boxplot(outlier.size = 0.0001,outlier.colour = "black")+
   labs(x = "",y = "expression (TPM)") +
   theme_classic() +
   theme(legend.position="bottom",
             panel.grid = element_blank(),
             axis.text.x = element_text(family = "sans",color = "black",size = 12,angle =
30,hjust = 1),
             axis.text.y = element_text(family = "sans",color = "black",size = 12),
             axis.title = element_text(family = "sans",color = "black",size = 14))
ggsave("GSE115002_raw_boxplot.jpeg",height=6,width=21)

GSE115002_expr_1 <- log2(GSE115002_expr[,-1]+1) %>% normalizeBetweenArrays() %>%
as.data.frame()
GSE115002_expr_1$geneID <- GSE115002_expr$geneID

ggplot(GSE115002_expr_1 %>%
melt(id.vars=c("geneID"),variable.name="Sample",value.name = "TPM"),
            aes(x = Sample,y = TPM)) +
   geom_boxplot(outlier.size = 0.0001,outlier.colour = "black")+
   labs(x = "",y = "expression (log2(TPM+1))") +
   theme_classic() +
   theme(legend.position="bottom",
             panel.grid = element_blank(),
             axis.text.x = element_text(family = "sans",color = "black",size = 12,angle =
30,hjust = 1),
             axis.text.y = element_text(family = "sans",color = "black",size = 12),
             axis.title = element_text(family = "sans",color = "black",size = 14))
ggsave("GSE115002_normalizeBetweenArrays_boxplot.jpeg",height=6,width=21)
write.csv(GSE115002_expr_1[,c(105,1:104)],"GSE115002_expr_log2TPM_normalizationBet
weenArrays.csv",
                quote=FALSE,row.names = FALSE)

GSE87340_TPM <- read.csv("../GSE87340_TPM_Final.csv",header=T,check.names = FALSE)
range(GSE87340_TPM[,-1])
GSE87340_TPM <- GSE87340_TPM[,colnames(GSE87340_TPM) %in%
c("geneID",All_sample_info$Sample)]
ggplot(GSE87340_TPM %>% melt(id.vars=c("geneID"),variable.name="Sample",value.name
= "TPM"),
            aes(x = Sample,y = log(TPM+1))) +
   geom_boxplot(outlier.size = 0.0001,outlier.colour = "black")+
   labs(x = "",y = "expression (log(TPM))") +
```

```
    theme_classic() +
    theme(legend.position="bottom",
                panel.grid = element_blank(),
                axis.text.x = element_text(family = "sans",color = "black",size = 12,angle =
30,hjust = 1),
                axis.text.y = element_text(family = "sans",color = "black",size = 12),
                axis.title = element_text(family = "sans",color = "black",size = 14))
ggsave("GSE87340_raw_boxplot.jpeg",height=6,width=21)

GSE87340_TPM_1 <- log2(GSE87340_TPM[,-1]+1) %>% normalizeBetweenArrays() %>%
as.data.frame()
GSE87340_TPM_1$geneID <- GSE87340_TPM$geneID
ggplot(GSE87340_TPM_1 %>%
melt(id.vars=c("geneID"),variable.name="Sample",value.name = "TPM"),
              aes(x = Sample,y = TPM)) +
    geom_boxplot(outlier.size = 0.0001,outlier.colour = "black")+
    labs(x = "",y = "expression (log2(TPM))") +
    theme_classic() +
    theme(legend.position="bottom",
                panel.grid = element_blank(),
                axis.text.x = element_text(family = "sans",color = "black",size = 12,angle =
30,hjust = 1),
                axis.text.y = element_text(family = "sans",color = "black",size = 12),
                axis.title = element_text(family = "sans",color = "black",size = 14))
ggsave("GSE87340_log2TPM_normalizeBetweenArrays_boxplot.jpeg",height=6,width=21)
dim(GSE87340_TPM_1)

write.csv(GSE87340_TPM_1[,c(47,1:46)],"GSE87340_expr_log2TPM_normalizationBetweenA
rrays.csv",
                    quote=FALSE,row.names = FALSE)

GSE140343_TPM <- read.csv("../GSE140343_TPM_Final.csv",header=T,check.names =
FALSE)

ggplot(GSE140343_TPM %>%
melt(id.vars=c("geneID"),variable.name="Sample",value.name = "TPM"),
              aes(x = Sample,y = log(TPM+1))) +
    geom_boxplot(outlier.size = 0.0001,outlier.colour = "black")+
    labs(x = "",y = "expression (log(TPM))") +
    theme_classic() +
    theme(legend.position="bottom",
                panel.grid = element_blank(),
                axis.text.x = element_text(family = "sans",color = "black",size = 12,angle =
30,hjust = 1),
```

```
            axis.text.y = element_text(family = "sans",color = "black",size = 12),
            axis.title = element_text(family = "sans",color = "black",size = 14))
ggsave("GSE140343_raw_boxplot.jpeg",height=6,width=21)

GSE140343_TPM_1 <- log2(GSE140343_TPM[,-1]+1) %>% normalizeBetweenArrays() %>%
as.data.frame()
GSE140343_TPM_1$geneID <- GSE140343_TPM$geneID

ggplot(GSE140343_TPM_1 %>%
melt(id.vars=c("geneID"),variable.name="Sample",value.name = "TPM"),
         aes(x = Sample,y = TPM)) +
  geom_boxplot(outlier.size = 0.0001,outlier.colour = "black")+
  labs(x = "",y = "expression (log2(TPM))") +
  theme_classic() +
  theme(legend.position="bottom",
            panel.grid = element_blank(),
            axis.text.x = element_text(family = "sans",color = "black",size = 12,angle =
30,hjust = 1),
            axis.text.y = element_text(family = "sans",color = "black",size = 12),
            axis.title = element_text(family = "sans",color = "black",size = 14))
ggsave("GSE140343_log2TPM_normalizeBetweenArrays_boxplot.jpeg",height=6,width=21)
dim(GSE140343_TPM_1)

write.csv(GSE140343_TPM_1[,c(101,1:100)],"GSE140343_expr_log2TPM_normalizationBet
weenArrays.csv",
              quote=FALSE,row.names = FALSE)


TCGA_LUAD_TPM <- read.csv("../TCGA_LUAD_TPM_Final.csv",header=T,check.names =
FALSE)
TCGA_LUAD_TPM <- TCGA_LUAD_TPM[,colnames(TCGA_LUAD_TPM) %in%
c("geneID",All_sample_info$Sample)]
dim(TCGA_LUAD_TPM)
ggplot(TCGA_LUAD_TPM %>%
melt(id.vars=c("geneID"),variable.name="Sample",value.name = "TPM"),
         aes(x = Sample,y = log(TPM+1))) +
  geom_boxplot(outlier.size = 0.0001,outlier.colour = "black")+
  labs(x = "",y = "expression (log(TPM))") +
  theme_classic() +
  theme(legend.position="bottom",
            panel.grid = element_blank(),
            axis.text.x = element_blank(),
            axis.text.y = element_text(family = "sans",color = "black",size = 12),
            axis.title = element_text(family = "sans",color = "black",size = 14))
```

```
ggsave("TCGA_LUAD_raw_boxplot.jpeg",height=6,width=40)

TCGA_LUAD_TPM_1 <- log2(TCGA_LUAD_TPM[,-1]+1) %>%
normalizeBetweenArrays() %>% as.data.frame()
TCGA_LUAD_TPM_1$geneID <- TCGA_LUAD_TPM$geneID
dim(TCGA_LUAD_TPM_1)
write.csv(TCGA_LUAD_TPM_1[,c(516,1:515)],"TCGA_LUAD_logTPM_normalizationBetweenA
rrays.csv",
                    quote=FALSE,row.names = FALSE)

TCGA_LUAD_TPM_1[1:5,1:5]
ggplot(TCGA_LUAD_TPM_1 %>%
melt(id.vars=c("geneID"),variable.name="Sample",value.name = "TPM"),
              aes(x = Sample,y = TPM)) +
    geom_boxplot(outlier.size = 0.0001,outlier.colour = "black")+
    labs(x = "",y = "expression (log(TPM))") +
    theme_classic() +
    theme(legend.position="bottom",
               panel.grid = element_blank(),
               axis.text.x = element_blank(),
               axis.text.y = element_text(family = "sans",color = "black",size = 12),
               axis.title = element_text(family = "sans",color = "black",size = 14))
ggsave("TCGA_LUAD_normalizationBetweenArrays_boxplot.jpeg",height=6,width=40)


dim(GSE115002_expr_1)
dim(GSE87340_TPM_1)
dim(GSE140343_TPM_1)
dim(TCGA_LUAD_TPM_1)


Express <- cbind(GSE115002_expr_1[,-105],
                                  GSE87340_TPM_1[,-47],
                                  GSE140343_TPM_1[,-101],
                                  TCGA_LUAD_TPM_1[,-516])

rownames(Express) <- GSE115002_expr_1$geneID
write.csv(Express,"All_sample_expression_log2TPM.csv",quote=FALSE)
# Express <- read.csv("All_sample_expression_log2TPM.csv",check.names =
FALSE,header=1,row.names = 1)
Express[1:5,1:5]

Express <- Express[,All_sample_info$Sample]
pca.plot(Express,factor(All_sample_info$DataSet))
```

```
dim(Express)

Express_tumor <- Express[,colnames(Express) %in%
All_sample_info[All_sample_info$Type=="Tumor",]$Sample]

setwd("./EMO/")
EMO <-
read.table("../04.Extracellular_matrix_organization/Extracellular_matrix_organization.txt",h
eader=FALSE)$V1
EMO
Express[1:5,1:5]
Express_EMO <- Express[EMO,] %>% as.data.frame()

Express_EMO$Gene <- rownames(Express_EMO)
Express_EMO.melt <- melt(Express_EMO,id.vars = c("Gene"),variable.name =
"Sample",value.name = "TPM") %>%
  left_join(All_sample_info,by="Sample")

head(All_sample_info)
anno <- All_sample_info[,c("Type","DataSet")]
rownames(anno) <- All_sample_info$Sample
unique(anno$DataSet)


## ECM in each dataset
library(pheatmap)
pheatmap(Express[,anno[anno$DataSet=="TCGA-LUAD",] %>%
dplyr::select(c("Type")) %>% dplyr::arrange(Type) %>% rownames()],
          annotation_col = anno[anno$DataSet=="TCGA-LUAD",] %>%
dplyr::select(c("Type")),
          color =
c(colorRampPalette(c("dodgerblue4","white"))(100),colorRampPalette(c("white","deeppink
4"))(100)),
          scale="row",
          treeheight_row = 0,
          cluster_cols = FALSE,
          show_rownames = FALSE,
          show_colnames = FALSE,
          filename = "00.TCGA-
LUAD_EMO_gene_heatmap.pdf",height=6,width=7,fontsize_row = 5)

pheatmap(Express[,anno[anno$DataSet=="GSE115002",] %>% dplyr::arrange(Type) %>%
dplyr::select(Type) %>% rownames()],
          annotation_col = anno[anno$DataSet=="GSE115002",] %>%
```

```
dplyr::select(c("Type")),
                  color =
c(colorRampPalette(c("dodgerblue4","white"))(100),colorRampPalette(c("white","deeppink
4"))(100)),
                  scale="row",
                  treeheight_row = 0,
                  cluster_cols = FALSE,
                  show_colnames = FALSE,
                  show_rownames = FALSE,
                  filename =
"00.GSE115002_EMO_gene_heatmap.pdf",height=6,width=7,fontsize_row = 5)

pheatmap(Express[,anno[anno$DataSet=="GSE140343",] %>% dplyr::arrange(Type) %>%
dplyr::select(Type) %>% rownames()],
                  annotation_col = anno[anno$DataSet=="GSE140343",] %>%
dplyr::select(c("Type")),
                  color =
c(colorRampPalette(c("dodgerblue4","white"))(100),colorRampPalette(c("white","deeppink
4"))(100)),
                  scale="row",
                  treeheight_row = 0,
                  cluster_cols = FALSE,
                  show_colnames = FALSE,
                  show_rownames = FALSE,
                  filename =
"00.GSE140343_EMO_gene_heatmap.pdf",height=6,width=7,fontsize_row = 5)
tmp <- Express[,anno[anno$DataSet=="GSE87340",] %>% dplyr::select(c("Type")) %>%
dplyr::arrange(Type) %>% rownames()]
pheatmap(tmp[rowSums(tmp)>0,],
                  annotation_col = anno[anno$DataSet=="GSE87340",] %>%
dplyr::select(c("Type")),
                  color =
c(colorRampPalette(c("dodgerblue4","white"))(100),colorRampPalette(c("white","deeppink
4"))(100)),
                  scale="row",
                  treeheight_row = 0,
                  cluster_cols = FALSE,
                  show_colnames = FALSE,
                  show_rownames = FALSE,
                  filename =
"00.GSE87340_EMO_gene_heatmap.pdf",height=6,width=7,fontsize_row = 5)

## EMO related Prognostic signature
clinical <-
```

```
read.csv("../04.Extracellular_matrix_organization/Sample_clinical_information.csv",header=
T)
colnames(clinical)[1] <- "DataSet"

Express_EMO_TCGA <- Express_EMO[,anno[anno$DataSet=="TCGA-LUAD" &
anno$Type=="Tumor",] %>%
                                  dplyr::select(c("Type")) %>%
                                  dplyr::arrange(Type) %>%
                                  rownames()]
Express_EMO_TCGA[1:10,1:10]

out <- matrix(nrow=0,ncol=7) %>% as.data.frame()
colnames(out) <-
c("gene","p.value","wald.test","beta","HR","HR.confint.lower","HR.confint.upper")
dim(Express_EMO_TCGA)
dim(clinical)
for(item in rownames(Express_EMO_TCGA)){
  res <- matrix(nrow=0,ncol=7)
  colnames(res) <-
c("gene","p.value","wald.test","beta","HR","HR.confint.lower","HR.confint.upper")

  Gene_expression = Express_EMO_TCGA[item,]

  merged_data = tibble(Sample = names(Gene_expression),
                       gene_expression = as.numeric(Gene_expression)) %>%
    dplyr::left_join(clinical, by = "Sample") %>%
    dplyr::filter(OS.day>0) %>%
    dplyr::select(Sample, gene_expression, OS.day, OS) %>%
    dplyr::rename(time = OS.day,
                  status = OS)

  fit <- coxph(Surv(time, status) ~ gene_expression, data = merged_data)
  x <-summary(fit)

  res$gene <- item
  res$p.value <- signif(x$waldtest['pvalue'][[1]],digits = 2)
  res$wald.test <- signif(x$waldtest['test'][[1]],digits = 2)
  res$beta <- signif(x$coefficients[1],digits = 3)
  res$HR <- signif(x$coefficients[2],digits = 3)

  res$HR.confint.lower <- signif(x$conf.int[,"lower .95"], 2)
  res$HR.confint.upper <- signif(x$conf.int[,"upper .95"],2)
  res <- as.data.frame(res)
  out <- rbind(out,res)
```

```
}
as.data.frame(out) %>% filter(p.value<0.01) %>% .[,1] %>% length()

Univariate_Cox <- out %>% filter(p.value<0.01) %>% as.data.frame()
write.csv(Univariate_Cox,"01.Univariate_cox_54_gene.csv",quote=FALSE,row.names = FALSE)
##
prognosis_signature_candidate <- out %>% filter(p.value<0.01) %>% .[,1]

df <- Express[prognosis_signature_candidate,]

df$Gene <- rownames(df)

## Progrostic model construction #####
## lasso 10 fold
library(glmnet)
clinical_TCGA <- clinical[clinical$DataSet=="TCGA-LUAD",] %>%
    filter(OS.day>0 & Type=="Tumor")
dim(clinical_TCGA)
candidate_exp <- Express[prognosis_signature_candidate,clinical_TCGA$Sample] %>%
    as.matrix() %>%
    t() %>%
    as.data.frame()
candidate_exp$Sample <- rownames(candidate_exp)
candidate_exp
data <- left_join(clinical_TCGA,candidate_exp,by="Sample") %>%
    na.omit() %>%
    filter(OS.day>0)
rownames(data) <- data$Sample
data
data$OS <- as.double(data$OS)
data$OS.day <- as.double(data$OS.day)
dim(data)
y <- data.matrix(Surv(time=data$OS.day,event= data$OS))
rownames(y) <- rownames(data)
dim(y)
str(y)

dim(data[,prognosis_signature_candidate])
dim(y)
data[,prognosis_signature_candidate]
library(glmnet)
fit <- glmnet(x=data[,prognosis_signature_candidate],y,family="cox",alpha=1)
fit
```

```
jpeg("02.LASSO_lambda.jpeg",height=300,width=350)
plot(fit,xvar="lambda",label=TRUE)
dev.off()

pdf("02.LASSO_lambda.pdf",height=3,width=3.5)
plot(fit,xvar="lambda",label=TRUE)
dev.off()

set.seed(1111)
lasso_fit <-
cv.glmnet(x=as.matrix(data[,prognosis_signature_candidate]),y,family="cox",nfold=10)
lasso_fit
jpeg("02.LASSO_lambda_1.jpeg",height=300,width=350)
plot(lasso_fit,label=T)
dev.off()

pdf("02.LASSO_lambda_1.pdf",height=3,width=3.5)
plot(lasso_fit,label=T)
dev.off()

coefficient <- coef(lasso_fit,s=lasso_fit$lambda.min)
Active.Index <- which(as.numeric(coefficient)!=0)
active.coeficient <- as.numeric(coefficient)[Active.Index]
sig_gene_multi_cox <- rownames(coefficient)[Active.Index]

multi_variate_cox <- coxph(Surv(OS.day, OS)~ B4GALT1+ FERMT1+ COL22A1+
                                            COL4A6 +
CSGALNACT1+FBN2+FGA+FOXC2+FSCN1+
                                            ITGA6+LOXL2+LAMB1+MYO1E,data= data)
ph_hypo_multi <- cox.zph(multi_variate_cox)
ph_hypo_table <- ph_hypo_multi$table[-nrow(ph_hypo_multi$table),]

formula_for_multivariate <-
as.formula(paste0('Surv(OS.day,OS)~',paste(rownames(ph_hypo_table)[ph_hypo_table[,3]>0
.05],sep='',collapse='+')))
multi_variate_cox2 <- coxph(formula_for_multivariate,data= data) ## remove FOXC2

correlation <-
cor(data[,rownames(ph_hypo_table)[ph_hypo_table[,3]>0.05]],method="pearson")
# install.packages("GGally")
library(GGally)

pdf("03.gene_correlation.pdf",height=12,width=12)
ggpairs(data[,rownames(ph_hypo_table)[ph_hypo_table[,3]>0.05]],axisLabels = "show")+
```

```
    theme_bw()+
    theme(panel.background = element_rect(color="black",size=1,fill = "white"),
            panel.grid = element_blank())
dev.off()

# install.packages('rms')
library('rms')
vif <- rms::vif(multi_variate_cox2)
sqrt(vif)<2

formula_for_multivariate <-
as.formula(paste0('Surv(OS.day,OS)~',paste(rownames(ph_hypo_table)[-
8],sep='',collapse='+')))
multi_variate_cox3 <- coxph(formula_for_multivariate,data= data)
ggforest(model = multi_variate_cox3,data=data,"Harzard ratio of candidate
genes",fontsize=0.8)
ggsave("04.forest_plot_12_gene.jpeg",height=6,width=10)
ggsave("04.forest_plot_12_gene.pdf",height=6,width=10)

## three gene COL4A6, FGA, FSCN1
multi_variate_cox4 <- coxph(Surv(OS.day, OS) ~ COL4A6 + FGA + FSCN1,data= data)
summary(multi_variate_cox4)
ggforest(model = multi_variate_cox4,data=data,"Harzard ratio of candidate
genes",fontsize=0.8)
ggsave("05.forest_plot_3_gene.jpeg",height=2,width=8)
ggsave("05.forest_plot_3_gene.pdf",height=2,width=8)
##
## estimation
## concordance index
C_index = multi_variate_cox4$concordance['concordance']
C_index # 0.68251
summary(multi_variate_cox4)

## ROC
library(dplyr)
summary(multi_variate_cox4)
signature <- c("COL4A6","FGA","FSCN1")

riskscore <- function(df,signature){
    risk_score_table <- df[,signature]
    for(each_sig_gene in colnames(risk_score_table)){
        risk_score_table$each_sig_gene <-
risk_score_table[,each_sig_gene]*(summary(multi_variate_cox4)$coefficients[each_sig_gene,
1])
```

```
    }
    risk_score_table <- cbind(risk_score_table,'total_risk_score'=
exp(rowSums(risk_score_table))) %>%
        cbind(data[,c("Sample","OS.day","OS")])
    risk_score_table<- risk_score_table[,c("Sample","OS.day","OS","total_risk_score")]
    risk_score_table
}
risk_score_table <- riskscore(data,signature)

library('survivalROC')
# install.packages('survivalROC')
multi_ROC <- function(time_vector,risk_score_table){
    single_ROC <- function(single_time){
        for_ROC <- survivalROC(Stime = risk_score_table$OS.day,
                                              status = risk_score_table$OS,
                                              marker = risk_score_table$total_risk_score,
                                              predict.time = single_time,method="KM")
        data.frame('True_positive'=for_ROC$TP,'False_positive'=for_ROC$FP,
                          'Cut_values' =
for_ROC$cut.values,'Time_point'=rep(single_time,length(for_ROC$TP)),
                          'AUC'=rep(for_ROC$AUC,length(for_ROC$TP)))

    }
    multi_ROC_list <- lapply(time_vector,single_ROC)
    do.call(rbind,multi_ROC_list)
}
for_multi_ROC <- multi_ROC(time_vector = c(365*seq(1,5,2)),risk_score_table =
risk_score_table)

# install.packages ("devtools")
# devtools::install_github('sachsmc/plotROC')
library(plotROC)
ggplot(for_multi_ROC,aes(x=False_positive,y=True_positive,label=Cut_values,color=as.factor(
Time_point)))+
    geom_roc(labels = F,stat="identity",n.cuts=0)+
    geom_abline(slope=1,intercept = 0,color="red",linetype=2)+
    theme_bw()+
    scale_color_manual(values=mypal[c(1,2,6)])+
    labs(x="FP",y="TP",color="")+
    theme(panel.background = element_rect(color="black",size=1,fill="white"),
              panel.grid=element_blank())+
    annotate("text",x=0.75,y=0.15,label=paste0("AUC max =
",round(max(for_multi_ROC$AUC),2) ))
ggsave("06.ROC_three_gene.pdf",height=3.5,width=5.5)
```

```
show_col(mypal)

##
library(pROC)
rocobj <- roc(risk_score_table$OS,risk_score_table$total_risk_score)
plot(rocobj,legacy.axes = TRUE,thresholds = "best",print.thres="best") # cutoff = 3.284

risk_score_table = risk_score_table %>%
    mutate(group = case_when(
        total_risk_score >= 3.284 ~ "High_risk",
        total_risk_score < 3.284 ~ "Low_risk",
        TRUE ~ NA_character_
    ))

table(risk_score_table$group)
fit = survfit(Surv(OS.day, OS) ~ group, data = risk_score_table)

p <- ggsurvplot(fit, data=risk_score_table, pval = TRUE,conf.int = FALSE,
                                ggtheme = theme_bw(),risk.table = TRUE,
                                risk.table.col = "strata",
                                palette = c("#E7B800", "#2E9FDF"),
                                break.x.by = 1000)
p

jpeg("07.TCGA_KM_final_three_gene.jpeg",width=375,height=400)
print(p)
dev.off()

pdf("07.TCGA_KM_final_three_gene.pdf",width=5,height=6)
print(p)
dev.off()

head(risk_score_table)
risk_score_table <- risk_score_table%>% arrange(total_risk_score)
head(risk_score_table)
risk_score_table$number <- 1:nrow(risk_score_table)
p1 <- ggplot(risk_score_table,aes(x=number,y=total_risk_score,color=group))+
    geom_point(size=0.5)+
    theme_bw()+
    scale_color_manual(values = c("#E7B800", "#2E9FDF"))+
    guides(colour = guide_legend(override.aes = list(size=3)))+
    labs(x="",y="Risk score",color="")+
    theme(legend.position = c(0.1,0.8))+
    geom_vline(xintercept = 311,linetype = 3)+
```

```
    theme(legend.key.size = unit(6,"pt"))
p1

p2 <- ggplot(risk_score_table,aes(x=number,y=OS.day,color=as.factor(OS)))+
    geom_point(size=1)+
    theme_bw()+
    scale_color_manual(values = mypal[c(3,8)],limits = c("0","1"),labels = c("Alive","Dead"))+
    guides(colour = guide_legend(override.aes = list(size=3)))+
    labs(x="",y="Survival time (days)",color="")+
    theme(legend.position = c(0.1,0.8))+
    geom_vline(xintercept = 311,linetype = 3)+
    theme(legend.key.size = unit(6,"pt"))

p1
p2

cowplot::plot_grid(p1,p2,nrow=2)
ggsave("08.Distribution_of_survival_status_and_Risk_score.pdf",height=4.5,width=8)

##pheatmap
head(risk_score_table)
rownames(risk_score_table) <- risk_score_table$Sample
pheatmap(t(data[risk_score_table$Sample,c("COL4A6","FGA","FSCN1")]),
              show_colnames = FALSE,
              treeheight_row = 0,
              cluster_cols = FALSE,
              annotation_colors = list(group=c(High_risk = "#E7B800",Low_risk=
"#2E9FDF")),
              scale = "row",
              color =
c(colorRampPalette(c("dodgerblue4","white"))(100),colorRampPalette(c("white","deeppink
4"))(100)),
              annotation_col = dplyr::select(risk_score_table,"group"),
              filename = "08.gene_expression_of_three_gene.pdf",height = 3,width=10)

## other three dataset
df <- Express_tumor[c("FGA","COL4A6","FSCN1"),] %>% as.matrix() %>% t() %>%
as.data.frame()
df$Sample <- rownames(df)
candidate_exp_other_3 <- left_join(clinical,df,by="Sample",all.y=TRUE) %>%
    filter(DataSet == "GSE115002" | DataSet =="GSE87340" | DataSet == "GSE140343") %>%
    na.omit()
candidate_exp_other_3$Risk_score <- 0.373786* candidate_exp_other_3$FGA +
    26.319070 *candidate_exp_other_3$COL4A6 + 2.321322*candidate_exp_other_3$FSCN1
```

```
head(candidate_exp_other_3)

#GSE115002
data_GSE115002 <- candidate_exp_other_3 %>% filter(DataSet=="GSE115002")
rocobj <- roc(data_GSE115002$OS,data_GSE115002$Risk_score)
plot(rocobj,legacy.axes = TRUE,thresholds = "best",print.thres="best") # cutoff = 73.171

data_GSE115002 = data_GSE115002 %>%
    mutate(group = case_when(
        Risk_score > 73.171 ~ "High_risk",
        Risk_score <= 73.171 ~ "Low_risk",
        TRUE ~ NA_character_
    ))
nrow(data_GSE115002)
fit_GSE115002 = survfit(Surv(OS.day, OS) ~ group, data = data_GSE115002)

p <- ggsurvplot(fit_GSE115002, data=data_GSE115002, pval = TRUE,conf.int = FALSE,
                ggtheme = theme_bw(),risk.table = TRUE,
                risk.table.col = "strata",
                palette = c("#E7B800", "#2E9FDF"))
p

pdf("09.GSE115002_KM_final_three_gene.pdf",width=5,height=6)
print(p)
dev.off()
# GSE87340
data_GSE87340 <- candidate_exp_other_3 %>% filter(DataSet=="GSE87340")
rocobj <- roc(data_GSE87340$OS,data_GSE87340$Risk_score)
plot(rocobj,legacy.axes = TRUE,thresholds = "best",print.thres="best") # cutoff = 3.260

data_GSE87340 = data_GSE87340 %>%
    mutate(group = case_when(
        Risk_score > 3.260 ~ "High_risk",
        Risk_score <= 3.260 ~ "Low_risk",
        TRUE ~ NA_character_
    ))
head(data_GSE87340)
fit_GSE87340 = survfit(Surv(OS.day, OS) ~ group, data = data_GSE87340)

p <- ggsurvplot(fit_GSE87340, data=data_GSE87340, pval = TRUE,conf.int = TRUE,
                ggtheme = theme_bw(),risk.table = TRUE,
                risk.table.col = "strata",
                palette = c("#E7B800", "#2E9FDF"),
                break.x.by=1000)
```

```
p

pdf("09.GSE87340_KM_final_three_gene.pdf",width=5,height=6)
print(p)
dev.off()

# GSE140343
data_GSE140343 <- candidate_exp_other_3 %>% filter(DataSet=="GSE140343")
rocobj <- roc(data_GSE140343$OS,data_GSE140343$Risk_score)
plot(rocobj,legacy.axes = TRUE,thresholds = "best",print.thres="best") # cutoff = 27.873

dim(data_GSE140343)
data_GSE140343 = data_GSE140343 %>%
   mutate(group = case_when(
      Risk_score >= 27.873 ~ "High_risk",
      Risk_score < 27.873 ~ "Low_risk",
      TRUE ~ NA_character_
   ))
head(data_GSE140343)
fit_GSE140343 = survfit(Surv(OS.day, OS) ~ group, data = data_GSE140343)

p <- ggsurvplot(fit_GSE140343, data=data_GSE140343, pval = TRUE,conf.int = TRUE,
                ggtheme = theme_bw(),risk.table = TRUE,
                risk.table.col = "strata",
                palette = c("#E7B800", "#2E9FDF"))
p

pdf("09.GSE140343_KM_final_three_gene.pdf",width=5,height=6)
print(p)
dev.off()

##
head(risk_score_table)
head(All_sample_info)
TCGA_sample_info <- filter(All_sample_info,DataSet=="TCGA-LUAD")
TCGA_risk_score_table <-
left_join(risk_score_table,TCGA_sample_info,by="Sample",all.x=TRUE)

dev.off()
dim(TCGA_risk_score_table)
TCGA_risk_score_table$Smoke_Status <- as.factor(TCGA_risk_score_table$Smoke_Status)
ggplot(TCGA_risk_score_table,aes(x=Smoke_Status,y=total_risk_score,fill=Smoke_Status)) +
   geom_boxplot(outlier.size = 0.3,width=0.5)+
   theme_classic()+
```

```
    labs(x="",y="Risk score")+
    scale_fill_manual(values = mypal[c(5,8)])+
    scale_x_discrete(labels = c("Non-smoker","Smoker"))+
    geom_signif(comparisons = list(c("0","1")),step_increase = 0.05,map_signif_level = T,
                          test = wilcox.test,size=0.2,textsize = 5,tip_length=0.01,color="black")+
    theme(legend.position = "none")
ggsave("10.risk_score_between_smoke_status.pdf",height = 4,width=3.5)

data1 <-
left_join(data,TCGA_risk_score_table[,c("Sample","total_risk_score","group")],by="Sample")
dim(data1)
rownames(data)
ggplot(data1,aes(x=as.factor(Smoke_Status),y=total_risk_score))+
    geom_violin()

ddply(data1,"Smoke_Status",summarise,mean=median(total_risk_score),number=length(Tu
mor_stage))


data1$Tumor_stage <- sub("A","",data1$Tumor_stage)
data1$Tumor_stage <- sub("B","",data1$Tumor_stage)
data2 <- data1

data2$total_risk_score

data2 = data2 %>%
    mutate(Risk_quantile = case_when(
        total_risk_score < quantile(total_risk_score, 0.25) ~ "1",
        total_risk_score >= quantile(total_risk_score, 0.25) & total_risk_score <
quantile(total_risk_score, 0.5) ~ "2",
        total_risk_score >= quantile(total_risk_score, 0.5) & total_risk_score <
quantile(total_risk_score, 0.75) ~ "3",
        total_risk_score > quantile(total_risk_score, 0.75) ~ "4",
        TRUE ~ NA_character_
    ))

data2$Smoke_Status <- sub("1","Smoker1",data2$Smoke_Status)
data2$Smoke_Status <- sub("0","Smoker2",data2$Smoke_Status)
data2 <- within(data2,{
    Risk_quantile <- factor(Risk_quantile,labels = c("1","2","3","4"))
})


cox5 <- coxph(Surv(OS.day, OS) ~ Age + Gender + Tumor_stage + Risk_quantile +
```

```
Smoke_Status,data= data2)
summary(cox5)
ggforest(cox5,data=data2,"Harzard ratio",fontsize=0.8)
ggsave("11.Hard_ratio.pdf",height=7,width=9)

## Nomogram construction ###
data3 <- data2
data3$TNM_stage[data3$Tumor_stage=="I"] <- "I"
data3$TNM_stage[data3$Tumor_stage=="II"] <- "II"
data3$TNM_stage[data3$Tumor_stage=="III"|data3$Tumor_stage=="IV"] <- "III/IV"
dd <- datadist(data3)
options(datadist="dd")

f <- cph(Surv(OS.day, OS) ~ Age+TNM_stage+total_risk_score,
          data= data3,
          x=TRUE,
          y= TRUE,
          surv=TRUE)

survival <- Survival(f)
survival1 <- function(x){survival(365,x)}
survival2 <- function(x){survival(1095,x)}
survival3 <- function(x){survival(1825,x)}

nom <- nomogram(f,fun = list(survival1,survival2,survival3),
                fun.at = seq(0.1,0.9,by=0.1),
                funlabel = c('1 year survival',
                             '3 year survival',
                             '5 year survival'))
pdf("12.Nonogram.pdf",height=7,width=10)
plot(nom)
dev.off()

summary(f)
cox6 <- coxph(Surv(OS.day, OS) ~ Age + TNM_stage + total_risk_score,data= data3)
summary(cox6)
cox7 <- coxph(Surv(OS.day, OS) ~ Age + TNM_stage,data= data3)
summary(cox7)

rownames(data3) <- data3$Sample
## AUC for multi_variate_cox4/ cox7/ cox6
library(dplyr)
summary(multi_variate_cox4)
head(data)
```

```
riskscore <- function(df,Pronosis_signature,fit){

    risk_score_table <- df[,Pronosis_signature]
    head(risk_score_table)
    for(each_sig_gene in colnames(risk_score_table)){
        each_sig_gene="TNM_stage"
        risk_score_table$each_sig_gene <-
risk_score_table[,each_sig_gene]*(summary(fit)$coefficients[each_sig_gene,1])

    }
    risk_score_table <- cbind(risk_score_table,'total_risk_score'=
exp(rowSums(risk_score_table))) %>%
        cbind(data[,c("Sample","OS.day","OS")])
    risk_score_table<- risk_score_table[,c("Sample","OS.day","OS","total_risk_score")]
    risk_score_table
}
risk_score_table

multi_ROC <- function(time_vector,risk_score_table){
    single_ROC <- function(single_time){
        for_ROC <- survivalROC(Stime = risk_score_table$OS.day,
                               status = risk_score_table$OS,
                               marker = risk_score_table$total_risk_score,
                               predict.time = single_time,method="KM")
        data.frame('True_positive'=for_ROC$TP,'False_positive'=for_ROC$FP,
                   'Cut_values' =
for_ROC$cut.values,'Time_point'=rep(single_time,length(for_ROC$TP)),
                   'AUC'=rep(for_ROC$AUC,length(for_ROC$TP)))

    }
    multi_ROC_list <- lapply(time_vector,single_ROC)
    do.call(rbind,multi_ROC_list)
}
for_multi_ROC <- multi_ROC(time_vector = c(365*seq(1,5,1)),risk_score_table =
risk_score_table)
cox4_AUC <- unique(for_multi_ROC[,c("Time_point","AUC")])

df_cox6 <- data3[,c("total_risk_score","Age","TNM_stage")]
df_cox6$signature_score <-
df_cox6[,"total_risk_score"]*(summary(cox6)$coefficients["total_risk_score",1])
df_cox6$Age_score <- df_cox6[,"Age"]*(summary(cox6)$coefficients["Age",1])
df_cox6_I <- filter(df_cox6,TNM_stage=="I")
df_cox6_I$stage_score <- 0
df_cox6_II <- filter(df_cox6,TNM_stage=="II")
```

```
df_cox6_II$stage_score <- summary(cox6)$coefficients["TNM_stageII",1]
df_cox6_III_IV <- filter(df_cox6,TNM_stage=="III/IV")
df_cox6_III_IV$stage_score <- summary(cox6)$coefficients["TNM_stageIII/IV",1]
df_cox6_risk_score_table <- rbind(df_cox6_I,df_cox6_II,df_cox6_III_IV)
df_cox6_risk_score_table$total_risk_score <- df_cox6_risk_score_table$signature_score +
    df_cox6_risk_score_table$Age_score+df_cox6_risk_score_table$stage_score
df_cox6_risk_score_table$Sample <- rownames(df_cox6_risk_score_table)

cox6_risk_score_table <- df_cox6_risk_score_table %>%
    left_join(data[,c("Sample","OS.day","OS")],by="Sample")
head(cox6_risk_score_table)
rownames(cox6_risk_score_table) <- cox6_risk_score_table$Sample

multi_ROC <- function(time_vector,risk_score_table){
    single_ROC <- function(single_time){
        for_ROC <- survivalROC(Stime = risk_score_table$OS.day,
                                                    status = risk_score_table$OS,
                                                    marker = risk_score_table$total_risk_score,
                                                    predict.time = single_time,method="KM")
        data.frame('True_positive'=for_ROC$TP,'False_positive'=for_ROC$FP,
                              'Cut_values' =
for_ROC$cut.values,'Time_point'=rep(single_time,length(for_ROC$TP)),
                              'AUC'=rep(for_ROC$AUC,length(for_ROC$TP)))

    }
    multi_ROC_list <- lapply(time_vector,single_ROC)
    do.call(rbind,multi_ROC_list)
}
cox6_for_multi_ROC <- multi_ROC(time_vector = c(365*seq(1,5,1)),risk_score_table =
cox6_risk_score_table)
cox6_AUC <- unique(cox6_for_multi_ROC[,c("Time_point","AUC")])

df_cox7 <- data3[,c("Age","TNM_stage")]
df_cox7$Age_score <- df_cox7[,"Age"]*(summary(cox7)$coefficients["Age",1])
df_cox7_I <- filter(df_cox7,TNM_stage=="I")
dim(df_cox7_I)
df_cox7_I$stage_score <- 0
df_cox7_II <- filter(df_cox7,TNM_stage=="II")
dim(df_cox7_II)
df_cox7_II$stage_score <- summary(cox7)$coefficients["TNM_stageII",1]
df_cox7_III_IV <- filter(df_cox7,TNM_stage=="III/IV")
dim(df_cox7_III_IV)

df_cox7_III_IV$stage_score <- summary(cox7)$coefficients["TNM_stageIII/IV",1]
```

```
df_cox7_risk_score_table <- rbind(df_cox7_I,df_cox7_II,df_cox7_III_IV)

df_cox7_risk_score_table$total_risk_score <- df_cox7_risk_score_table$Age_score +
    df_cox7_risk_score_table$stage_score
dim(df_cox7_risk_score_table)
df_cox7_risk_score_table$Sample <- rownames(df_cox7_risk_score_table)

cox7_risk_score_table <- df_cox7_risk_score_table %>%
    left_join(data3[,c("Sample","OS.day","OS")],by="Sample")
head(cox7_risk_score_table)
rownames(cox7_risk_score_table) <- cox7_risk_score_table$Sample

cox7_for_multi_ROC <- multi_ROC(time_vector = c(365*seq(1,5,1)),risk_score_table =
cox7_risk_score_table)

cox7_AUC <- unique(cox7_for_multi_ROC[,c("Time_point","AUC")])
dim(cox7_for_multi_ROC)
dim(cox6_for_multi_ROC)
dim(for_multi_ROC)

cox4_AUC$Type <- "signature_only"
cox6_AUC$Type <- "combined"
cox7_AUC$Type <- "clinical_only"

AUC <- rbind(cox4_AUC,cox6_AUC,cox7_AUC)
AUC$year <- AUC$Time_point/365
AUC$Type
ggplot(AUC,aes(x=year,y=AUC,color=Type))+
    geom_point()+
    geom_line()+
    theme_bw()+
    scale_color_manual(values = c("#DC0000B2","#00A087B2","#3C5488B2"),
                                            limits = c("clinical_only","signature_only","combined"),
                                            labels =
c("clinical_model","signature_model","combined_model"))+
    labs(x="Time (year)",y="Time dependent AUC",color="")+
    scale_y_continuous(limits = c(0.5,0.8))+
    theme(legend.position = c(0.1,0.2))
ggsave("13.Time_dependent_ROC_curve.pdf",height=4,width=8)

## calibration
f1 <- cph(Surv(OS.day, OS) ~ Age+TNM_stage+total_risk_score,
                    data= data3,
                    x=TRUE,
```

```
                    y= TRUE,
                    surv=TRUE,
                    time.inc = 365)
cal1<-calibrate(f1, cmethod="KM", method="boot",u=365,m=100,B=1000)

f2 <- cph(Surv(OS.day, OS) ~ Age+TNM_stage+total_risk_score,
                    data= data3,
                    x=TRUE,
                    y= TRUE,
                    surv=TRUE,
                    time.inc = 730)
cal2<-calibrate(f2, cmethod="KM", method="boot",u=730,m=100,B=1000)

f3 <- cph(Surv(OS.day, OS) ~ Age+TNM_stage+total_risk_score,
                    data= data3,
                    x=TRUE,
                    y= TRUE,
                    surv=TRUE,
                    time.inc = 1095)
cal3<-calibrate(f3, cmethod="KM", method="boot",u=1095,m=100,B=1000)

f4 <- cph(Surv(OS.day, OS) ~ Age+TNM_stage+total_risk_score,
                    data= data3,
                    x=TRUE,
                    y= TRUE,
                    surv=TRUE,
                    time.inc = 1460)
cal4<-calibrate(f4, cmethod="KM", method="boot",u=1460,m=100,B=1000)

f5 <- cph(Surv(OS.day, OS) ~ Age+TNM_stage+total_risk_score,
                    data= data3,
                    x=TRUE,
                    y= TRUE,
                    surv=TRUE,
                    time.inc = 1825)
cal5<-calibrate(f5, cmethod="KM", method="boot",u=1825,m=100,B=1000)

pdf("Calibration.pdf",height = 6,width=7)
plot(cal1,lwd = 2,lty = 0,errbar.col = c("#2166AC"),
          bty = "l",
          xlim = c(0,1),ylim= c(0,1),
          xlab = "Nomogram-prediced OS (%)",ylab = "Observed OS (%)",
          col = c("#2166AC"),
          cex.lab=1.2,cex.axis=1, cex.main=1.2, cex.sub=0.6)
```

```
lines(cal1[,c('mean.predicted',"KM")],
            type = 'l', lwd = 1, col = c("#2166AC"), pch = 1)
mtext("")

plot(cal3,lwd = 2,lty = 0,errbar.col = mypal[1],
          xlim = c(0,1),ylim= c(0,1),col = mypal[1],add = T)
lines(cal3[,c('mean.predicted',"KM")],
            type = 'b', lwd = 1, col = mypal[1], pch = 16)


plot(cal5,lwd = 2,lty = 0,errbar.col = mypal[3],
          xlim = c(0,1),ylim= c(0,1),col = mypal[3],add = T)
lines(cal5[,c('mean.predicted',"KM")],
            type = 'b', lwd = 1, col = mypal[3], pch = 16)

abline(0,1, lwd = 2, lty = 3, col = c("#224444"))

legend(x=0.1,y=0.8,
             legend = c("1-year","3-year","5-year"),
             col =c("#2166AC",mypal[1],mypal[3]),
             lwd = 2,
             cex = 1,
             bty = "n")
dev.off()

## statistics
head(data3)
df <- table(data3$Tumor_stage,data3$group) %>% as.data.frame()
ggplot(df,aes(x=Var2,y=Freq,fill=Var1))+
  geom_col(position = "fill",width=0.6)+
  theme_classic()+
  labs(x="",y="Relative abundence (%)",fill="")+
  scale_y_continuous(expand=c(0,0),labels = scales::percent)+
  # scale_x_discrete(limits = c("Low_risk","High_risk"),labels = c("Low risk","High risk"))+
  scale_fill_manual(values = mypal[c(2,3,1,4)],labels = c("Satge I","Stage II","Stage III","Stage
IV"))
ggsave("15.Stage_distribution_between_risk_group.pdf",height=4,width=4)
```
